# Rhizoma Atractylodis Macrocephalae—Assessing the influence of herbal processing methods and improved effects on functional dyspepsia

**DOI:** 10.3389/fphar.2023.1236656

**Published:** 2023-08-04

**Authors:** Song-Hong Yang, Jing Zhu, Wen-Ting Wu, Jun-Mao Li, Heng-Li Tong, Yi Huang, Qian-Feng Gong, Fei-Peng Gong, Ling-Yun Zhong

**Affiliations:** ^1^ School of Pharmacy, Jiangxi University of Chinese Medicine, Nanchang, China; ^2^ Jiangxi Provincial People’s Hospital, The First Affiliated Hospital of Nanchang Medical College, Nanchang, China

**Keywords:** herbal processing method, Rhizoma Atractylodis Macrocephalae, network analysis, functional dyspepsia, ethnopharmacology

## Abstract

**Background:** The unique pharmaceutical methods for the processing of botanical drugs according to the theory of traditional Chinese medicine (TCM) affect clinical syndrome differentiation and treatment. The objective of this study was to comprehensively elucidate the principles and mechanisms of an herbal processing method by investigating the alterations in the metabolites of Rhizoma Atractylodis Macrocephalae (AMR) processed by Aurantii Fructus Immaturus (AFI) decoction and to determine how these changes enhance the efficacy of aqueous extracts in treating functional dyspepsia (FD).

**Methods:** A qualitative analysis of AMR before and after processing was conducted using UPLC-Q-TOF-MS/MS, and HPLC was employed for quantitative analysis. A predictive analysis was then conducted using a network analysis strategy to establish a botanical drug–metabolite–target–disease (BMTD) network and a protein–protein interaction (PPI) network, and the predictions were validated using an FD rat model.

**Results:** A total of 127 metabolites were identified in the processed AMR (PAMR), and substantial changes were observed in 8 metabolites of PAMR after processing, as revealed by the quantitative analysis. The enhanced aqueous extracts of processed AMR (PAMR) demonstrate improved efficacy in treating FD, which indicates that this processing method enhances the anti-inflammatory properties and promotes gastric motility by modulating DRD2, SCF, and c-kit. However, this enhancement comes at the cost of attenuating the regulation of motilin (MTL), gastrin (GAS), acetylcholine (Ach), and acetylcholinesterase (AchE).

**Conclusion:** Through this series of investigations, we aimed to unravel the factors influencing the efficacy of this herbal formulation in improving FD in clinical settings.

## 1 Introduction

The unique pharmaceutical methods for the processing of botanical drugs according to the theory of traditional Chinese medicine (TCM) affect clinical syndrome differentiation and treatment. These methods are very similar to cooking because both of these processes involve the use of natural botanical drugs and spices, such as vinegar, salt, and honey. These methods also reflect the theory that humans are an integral part of nature through the combination of diet and therapeutic thinking. Processing can cause changes in different botanical drugs, such as enhancing their efficacy, reducing their toxicity, or changing their site of action (Chen et al., 2020; Shan et al., 2020).

Rhizoma Atractylodis Macrocephalae (AMR) is the dried mature rhizome of *Atractylodes macrocephala* Koidz and has been used for thousands of years to treat a variety of diseases, including hyposplenic function, anorexia, abdominal distension, diarrhea, dizziness and palpitations (Huang et al., 2016; Shu et al., 2017). As the most important immune-enhancing botanical drug among classical Chinese medicines, AMR also exhibits a variety of biological properties, including gastrointestinal (GI) function improvement and antitumor, anti-inflammatory, antiaging, antioxidant, anti-osteoporosis, antibacterial and neuroprotective activities. Aurantii Fructus Immaturus (AFI) is the dried immature young fruit of *Citrus aurantium* L. and has been a commonly used botanical drug for thousands of years. Similar to many citrus botanical drugs, such as Chenpi (the peel of *Citrus reticulata* Blanco) and Foshou (the fruit of *Citrus medica* ‘Fingered’), AFI is also used as a flavoring agent in foods and has health benefits when consumed regularly. In addition, AFI is pickled with chili peppers and eaten as an appetizer in Jiangxi Province, China. AFI is mainly used as an adjuvant for the treatment of indigestion, loss of appetite, abdominal distension, abdominal pain, chest pain, organ prolapse and other diseases (Gao et al., 2021).

Functional dyspepsia (FD) is a nonorganic disorder of the upper GI tract that presents with painful or uncomfortable sensations that can significantly affect a patient’s mood and reduce their quality of life while causing dietary discomfort. However, the currently marketed FD drugs, such as proton pump inhibitors, tricyclic antidepressants and prokinetic drugs, are not particularly satisfactory (Quigley, 2017). Excitingly, TCM has some unique features that can be exploited for the treatment of FD because in Chinese medicine theory, the symptoms of FD are equivalent to the TCM term “stuffiness and fullness,” which makes AMR and AFI especially suitable for FD treatment. The combination of AMR and AFI is a classic TCM formula for the treatment of stomach diseases, especially FD (Wang et al., 2012). Consistent with this, raw Rhizoma *Atractylodis macrocephalae* (RAMR) can be processed with an AFI decoction by stir-frying to produce the final product “AMR processed by Aurantii Fructus Immaturus decoction” (PAMR). This processing method combines the functions of AMR and AFI into the final product, which is more effective and convenient to use than AMR. However, no comprehensive and systematic study has investigated why such a processing method should be conducted. Botanical drugs and their metabolites always have multiple targets. Evaluating the curative effect of herbal medicines is also challenging because these medicines are composed of many bioactive metabolites. Moreover, research on pre- and postprocessing makes the study of TCMs more complicated. Therefore, a comprehensive explanation of these issues is a major challenge. In recent years, network analysis has been widely used to explain the therapeutic effects and targets of TCMs and their potentially active metabolites. The concept of “network targets, multiple metabolites” in network analysis is the most suitable tool for investigating the efficacy of TCMs at the molecular level (Zhang et al., 2013a). In preliminary work conducted by our laboratory, we used network analysis to reveal the mechanisms of action of some botanical drugs, especially those with dual uses as medicine and food (Wang et al., 2020b; Wu et al., 2020; Wu et al., 2021; Xiao et al., 2023). In this study, ultrahigh-performance liquid chromatography coupled with quadrupole time-of-flight tandem mass spectrometry (UPLC–Q–TOF–MS/MS) and high-performance liquid chromatography (HPLC) methods were utilized for qualitative analysis and determination of the contents in the samples before and after processing. An integrated method combining quantitative comparisons with an untargeted metabolomics approach was used to verify the chemical differences between the samples. Subsequently, various networks, including a botanical drug–metabolite–target–disease (BMTD) network and a protein–protein interaction (PPI) network, were established for predictive analysis, and these preliminary results were confirmed in subsequent *in vivo* experiments. This study aimed to provide an initial clarification of the mechanisms of processed AMR (PAMR), a processing method used for ancient classical Chinese medicines, and to offer references for the preservation and inheritance of other herbal pharmaceutical technologies recorded in ancient books, the underlying principles of which remain unclear. Through this series of investigations, we aimed to unravel the factors underlying the efficacy of this herbal formulation in improving FD in clinical settings.

## 2 Materials and methods

### 2.1 Chemicals and materials

*A. macrocephala* Koidz rhizomes constituted the raw material of the collected AMR samples (RAMRs). All fresh RAMRs of *A. macrocephala* Koidz were picked in December 2019 (No. 20191215) in Shiliang Town, Tiantai County, Zhejiang Province, China (E121° 9′15″, N29° 13′26″). Dried immature young fruit of *Citrus aurantium* L. was the raw material of the collected AFI samples (RAFIs, No. 20190621), which were purchased from Jiangxi Gu Han Refined Chinese Medicine Decoction Pieces Co., Ltd. (Nanchang, China). According to our previous research and processing technology optimization (Wang et al., 2018), we generated Rhizoma *Atractylodis macrocephalae* processed by Aurantii Fructus Immaturus decoction (PAMR, No. 20191225). In brief, 500 mL of AFI decoction (obtained by adding 100 g of AFI to 1430 mL of pure water, extracting at 100°C for 90 min, filtering, and adding to 1200 mL of pure water for extraction at 100°C for 90 min; the extracts were combined and concentrated to 500 mL with occasional turning) was added to 1000 g of AMR. After soaking for 24 h, the sample was stir-fried at 110°C for 6 min and then dried at 40°C for 24 h. All materials were identified by Professor Lingyun Zhong of Jiangxi University of Chinese Medicine and stored in the Department of TCM Processing, Jiangxi University of Chinese Medicine.

Naringenin (lot no. CHB180916), hesperidin (lot no. CHB180523), diosmin (lot no. CHB180125), hesperetin (lot no. CHB180524), tangeretin (lot no. CHB190125), neohesperidin (lot no. CHB180316), 4′,5,7-trimethoxyflavone (lot no. CHB181127), 5,7,3′-trihydroxy-6,4′,5′-trimethoxyflavone (lot no. CHB190311), 5-hydroxy-3′,4′,6,7,8-pentamethoxyflavone (lot no. CHB180321), narirutin (lot no. CHB180917), neoeriocitrin (lot no. CHB180311), naringenin-7-O-glucoside (lot no. CHB181105), naringin (lot no. CHB180914), 3,3′,4′,5,6,7,8-heptamethoxyflavone (lot no. CHB180120), limonin (lot no. CHB180124), eriocitrin (lot no. CHB190219), poncirin (lot no. CHB180625), and vicenin II (lot no. CHB180322) were purchased from Chengdu Chroma-Biotechnology Co., Ltd. (Chengdu, China). Synephrine (lot no. MUST19101716), atractylenolide Ⅰ (lot no. MUST19030221), atractylenolide II (lot no. MUST19101113), atractylenolide III (lot no. MUST19101114) and atractylone (lot no. MUST20070305) were purchased from Chengdu Manst Biotechnology Co., Ltd. (Chengdu, China). The purities of all chemical standards were ≥98%. Water for HPLC and UPLC–Q–TOF–MS/MS analyses was purified using a Milli-Q system (Millipore, Bedford, MA, United States). Iodoacetamide (IAA) was purchased from Sigma‒Aldrich Chemicals (St. Louis, Missouri, United States). Domperidone was obtained from Xi’an Janssen Pharmaceutical Ltd. (Xi’an, China). All other chemicals were of analytical or chromatographic grade.

### 2.2 Sample preparation

To determine the contents of different standard metabolites in RAMR, RAFI, and PAMR, the samples were crushed and passed through a 200-mesh sieve. In a 50-mL centrifuge tube, 1.00 g of each sample powder was accurately weighed, and 10 mL of methanol solution was added. Each mixture was then extracted for 10 min at 25°C with ultrasonication and sequentially centrifuged for 5 min at 8000 r·min^−1^ at 25°C. The extraction procedure was repeated once. The supernatants were combined, concentrated, and diluted to 10 mL with methanol. The final extraction solution was filtered through a 0.22-μm nylon 66 microfiltration membrane before HPLC analysis. Six replicates of each sample were prepared in parallel.

To explore the metabolites in the different samples, 1.00 g of sample powder was extracted for 30 min using 25 mL of methanol with ultrasonication. After the samples were cooled to room temperature (25°C), methanol was added to compensate for the weight loss before centrifugation for 10 min at 8000 r·min^−1^. Subsequently, 1 mL of the supernatant was collected, diluted to 10 mL with methanol and filtered through a 0.22-μm nylon 66 microfiltration membrane. Ten microliters of each sample was placed in a vial to generate a mixed quality control (QC) sample to validate the reproducibility and stability of the UPLC–Q–TOF–MS/MS method.

### 2.3 Qualitative analysis of the metabolites in the samples

UPLC analysis was conducted using an Agilent 1290 LC system (Agilent Technologies, Palo Alto, CA, United States) equipped with an automatic degasser, an infinity binary pump, and an autosampler. A Waters ACQUITY UPLC CSHTM C18 column (2.1 × 100 mm, 1.7 μm; Waters Corp.) was utilized for chromatographic separation. The mobile phases consisted of 0.1% formic acid/water (A) and acetonitrile (B). The mobile phase gradient was as follows: 0–1 min, 5% B; 1–5 min, 5%–10% B; 5–10 min, 10%–18% B; 10–22 min, 18%–24% B; 22–30 min, 24%–45% B; 30–41 min, 45%–95% B; 41–41.5 min, 95%–5% B; and 41.5–45 min, 5% B. The flow rate was set to 0.30 mL·min^−1^, the injection volume was 2 μL, the column oven was set to 40°C, and the automatic injector temperature was set to 4°C.

MS/MS detection was conducted using a time-of-flight mass spectrometer (Triple TOF^TM^ 5600+ system) equipped with a Duo Spray source for detecting ions in both the positive and negative modes with high resolution (AB SCIEX, Foster City, CA, United States). In the positive mode, electrospray ionization was applied with the following parameters: ion spray voltage, 5500 V; ion source temperature, 500°C; curtain gas, 40 psi; nebulizer gas (GS 1), 50 psi; heater gas (GS 2), 50 psi; and declustering potential (DP), 100 V. In the information-dependent acquisition (IDA) experiment, the collision energy (CE) was set to 35 eV, and the collision energy spread (CES) was ±10 eV. In the negative mode, electrospray ionization was applied with the following parameters: ion spray voltage, −4500 V; ion source temperature, 550°C; curtain gas, 40 psi; GS 1, 50 psi; GS 2, 50 psi; and DP: −100 V. In the IDA experiment, the CE was set to −35 eV, and the CES was ±10 eV. In both the positive and negative ion modes, the mass ranges were m/z 50–1250 Da for the TOF−MS and MS/MS scans. MS/MS fragmentation was selected for the eight ions with the most intense signals from each TOF−MS scan. Dynamic background subtraction (DBS) was applied to match the IDA tests for UPLC−Q−TOF−MS/MS. All gases were high-purity nitrogen.

To enhance the analysis for precise metabolite identification, UPLC-Q-TOF-MS/MS analysis was performed using 23 standard metabolites. The same LC−MS-related parameters were used to ensure consistent ion cleavage.

### 2.4 Method validation of qualitative analysis

To ensure the reliability of the method used, verification was conducted by analyzing QC samples prior to sample analysis. Twenty-three chromatographic peaks were randomly selected from the chromatograms of the QC samples, and the relative standard deviations (RSDs) of the peak area and retention time (RT) were calculated to evaluate the precision, repeatability, and sample stability of the system. Each QC sample was tested 6 times to determine the precision of the system. The same QC sample was prepared six times in parallel, and the repeatability of the method was evaluated after all injections were complete. In addition, the stability of the samples was investigated by injecting and analyzing the QC samples at 0, 6, 12, 18, and 24 h.

### 2.5 Quantitative analysis of the metabolites of the samples

The samples were quantitatively analyzed using a Waters 2695 HPLC system equipped with a 2996 PDA detector (Waters, Milford, MA, United States). For the separation of synephrine, a Titank C18 column (250 × 4.6 mm, 5 μm; Waters, Milford, MA, United States) was used. The HPLC settings for synephrine were as follows: injection volume, 10 μL; column oven, 30°C; flow rate, 1.0 mL·min^−1^; and detection wavelength, 275 nm. Mobile phases A and B were methanol and potassium phosphate dibasic aqueous solutions (0.6 g of potassium phosphate dibasic, 1.0 g of sodium laurylsulfonate, and 1.0 mL of acetate dissolved in water and diluted to 1000 mL), respectively, and elution was conducted isocratically (50%–50%) for 20 min. For analysis, synephrine was precisely weighed and dissolved in methanol. The synephrine standard solution was then diluted to different concentrations (1000, 500, 250, 100, 50, and 25 μg·mL^−1^) before HPLC analysis. The HPLC settings for the other 7 metabolites (atractylenolide Ⅰ, atractylenolide II, atractylenolide III, atractylone, neohesperidin, hesperidin and naringin) were as follows: injection volume, 10 μL; column oven temperature, 30°C; flow rate, 1.0 mL·min^−1^; and detection wavelengths, 275 nm (atractylenolide II, atractylenolide III and atractylone), 275 nm (atractylenolide Ⅰ), and 283 nm (neohesperidin, hesperidin and naringin). Mobile phases A and B were methanol and water, respectively. Gradient elution was performed using the following program: 0–50 min, 5% A-50% A; 50–85 min, 50% A-80% A; and 85–120 min, 80% A-95% A. For analysis, these 7 metabolites were precisely weighed and dissolved in methanol. The standard solutions were diluted to different concentrations (100, 50, 25, 10, 5, and 2.5 μg·mL^−1^) before HPLC analysis.

### 2.6 Screening of potentially active metabolites

Using UPLC-Q-TOF-MS/MS analysis, a database of PAMR metabolites considered potentially active metabolite candidates was established. Subsequently, the activity of these metabolites was screened. Based on previous experience from relevant studies (Yang et al., 2020), two classic ADME parameters, namely, oral bioavailability (OB) and drug likeness (DL), were used as screening strategies in this study. OB refers to the percentage of unmodified drug that enters the circulation after oral administration (Liu et al., 2013). In this study, a nonlinear support vector regression (SVR) mathematical model was constructed to predict the OB (Xu et al., 2012). The final expression of the SVR function is as follows: $f\left(\alpha \right)=\sum_{i=1}^{n}\left(A_{i}-A_{i}^{*}\right)K\left(\alpha ,\alpha_{i}\right)+B$ . The Lagrange multipliers *A*_*i*_ and *A*_i_^*^ are obtained by minimizing the canonical risk function. *B* is the regression parameter. Moreover, its kernel function *K*(*α*, *α*_*i*_) is obtained from the linear dot product of the nonlinear mapping using the following formula: $K\left(\alpha ,\alpha_{i}\right)=\phi \left(\alpha \right)\cdot \phi \left(\alpha_{i}\right)$ . A higher value indicates a higher possibility that the metabolite could be used in clinical practice (Alam et al., 2015). DL can predict the likelihood of a metabolite becoming a drug by comparing the functional groups of the target metabolite with the active functional groups that exist in known drugs (Walters and Murcko, 2002). In this study, the Tanimoto coefficient was used to evaluate the DL index of the PAMR metabolites with the following specific formula: $T\left(\alpha ,\beta \right)=\frac{\alpha \times \beta }{\alpha^{2}+\beta^{2}-\alpha \times \beta }$ , where *α* was calculated using Dragon V7.0 software (chm.kode-solutions.net/products_dragon.php) based on the molecular properties of metabolites in the prepared samples of PAMR and β is the average molecular properties of all drugs in the DrugBank database (www.drugbank.ca/) (Mauri et al., 2006). Metabolites with OB ≥ 15% and DL ≥ 0.10 were considered to have relatively good pharmacological properties.

### 2.7 Prediction of targets related to the potentially active metabolites of PAMR

Because TCMs have modes of action that occur through multiple metabolites and targets, the prediction of potential targets is particularly important. Computer models based on large datasets, which are currently relatively mature, were utilized to predict potential interactions between the drugs and targets in this study. The model integrated chemical, genomic and pharmacological information with a core composed of random forest (RF) and support vector machine (SVM) methods. Because these are well-known and widely used theories, only brief descriptions are given here (Breiman, 2001; Burbidge et al., 2001; Zhao et al., 2006). This study used the R randomForest function in the software package (http://cran.r-project.org/web/packages/randomForest/index.html, Publisher on 25 March 2018) to perform classification and regression, optimize the tuning parameters ntree and mtry to eliminate redundant descriptors that would seriously affect the performance of the model, and use out-of-bag (OOB) errors to obtain feature importance estimates (Hao et al., 2010; Hao et al., 2011). For nonlinearly separated data, an SVM method based on the radial basis function (RBF) was used for classification according to the following formula: $f\left(x\right)=sign\left[\sum_{i=1}^{n}\alpha y_{i}k\left(x,x_{i}\right)+\beta \right]$ , where x_i_ is a set of descriptors; y_i_ is the input class label with a value of −1 or 1; *f*(*x*) is the decision function; and k (x,x_i_) is a kernel function that shows the similarity between two vectors, which can be obtained from the linear dot product of nonlinear maps, *k*(*x*, *x*_*i*_) = *φ*(*x*)·*φ*(*x*_*i*_). The parameters *α* and *β* can be obtained by solving the quadratic programming problem. According to the positive or negative value of the decision function, the relationships between the metabolites and proteins were classified as positive or negative, respectively. A positive sample indicates that a metabolite interacts with a protein; a negative sample indicates that a metabolite interacts with a protein in a nonproductive manner. The R kernlab function in the software package (https://cran.rstudio.com/web/packages/kernlab/index.html, 12 November 2019) was then used to establish the SVM classification model.

### 2.8 Acquisition of FD-Related targets

The keyword “functional dyspepsia” was used to collect the related targets from two sources in this study. The first source was GeneCards®: The Human Gene Database V5.0.0 (www.genecards.org/, updated on 22 September 2020), and the second source was the Online Mendelian Inheritance in Man (OMIM) Database (www.omim.org/, updated on 28 February 2019). In addition, to collect the relevant targets of FD as comprehensively as possible, we also reviewed the literature.

### 2.9 Construction of the botanical drug–metabolite–target–disease (BMTD) network

The possible association between PAMR and FD was analyzed by crossing the predicted potentially active metabolites with the acquired disease-related targets, resulting in a Venn diagram of the common targets. The BMTD network was then established and visualized using Cytoscape software V3.7.1 (www.cytoscape.org/).

### 2.10 Generation of the PPI network

The STRING online database (https://string-db.org/; the organism was set to *Homo sapiens*) was used to obtain the PPI data of the molecular targets of PAMR. Subsequently, the PPI network was established, and a topological analysis was performed.

### 2.11 GO and KEGG pathway enrichment analyses

To more deeply analyze why PAMR can treat FD, we performed GO and KEGG pathway enrichment analyses using Bioconductor (R) V3.8 bioinformatics software (http://bioconductor.org/). GO items (*p.*adjust ≤ 0.05) were collected for functional annotation clustering. The KEGG database was used for pathway enrichment analysis to verify statistically significant gene functional categories (*p.*adjust ≤ 0.05).

### 2.12 Preparation of botanical drug aqueous extract

RAMR, RAFI, and PAMR, each weighing 1000 g, were soaked in a tenfold volume of water for 30 min before decoction. The mixture was brought to a boil and maintained at a low boil for 30 min. The aqueous extracts were then filtered out, and the process was repeated once more (maintaining a low boil for 30 min after boiling). All aqueous extracts were combined, filtered, concentrated and dried with a Freeze Dryer (EYELA, Japan). The yields of the powdered aqueous extracts were 67.3% for RAMR, 38.1% for RAFI and 68.61% for PAMR.

### 2.13 Animal experiments

Ten-day-old specific pathogen-free (SPF) male Sprague‒Dawley (SD) rat pups were obtained from the Anshengmei Pharmaceutical Research Institute Co., Ltd. (SYXK 2018-0004, Changsha, China). These animals were randomly divided into a normal control (NC) group and an FD model group. The ten-day-old rat pups in the NC group were given 0.2 mL of 2% sucrose solution daily for 6 days, and the ten-day-old rat pups in the FD model group were given 0.2 mL of a mixed solution of 0.1% IAA and 2% sucrose daily for 6 days to establish the FD model (Wei et al., 2019). When the IAA-treated rats were 6 weeks of age, they were fed every other day for 2 weeks of alternate-day fasting. Starting from the eighth week, the model rats were further divided into treatment groups and administered the appropriate combination of aqueous extracts as follows: low-dosage RAMR (RAMR-L, 0.63 g·kg^−1^ daily, *n* = 10), medium-dosage RAMR (RAMR-M, 1.26 g·kg^−1^ daily, equivalent to a clinical daily dosage of 12 g for a 60-kg adult, *n* = 10), and high-dosage RAMR (RAMR-H, 2.52 g·kg^−1^ daily, *n* = 10); low-dosage PAMR (PAMR-L, 0.63 g·kg^−1^ daily, *n* = 10), medium-dosage PAMR (PAMR-M, 1.26 g·kg^−1^ daily, equivalent to a clinical daily dosage of 12 g for a 60-kg adult, *n* = 10), and high-dosage PAMR (PAMR-H, 2.52 g·kg^−1^ daily, *n* = 10); low-dosage RAFI (RAFI-L, 0.063 g·kg^−1^ daily, *n* = 10), medium-dosage RAFI (RAFI-M, 0.126 g·kg^−1^ daily, equivalent to a clinical daily dose of 1.2 g for a 60-kg adult, *n* = 10), and high-dosage RAFI (RAFI-H, 0.252 g·kg^−1^ daily, *n* = 10); and domperidone (3.15 mg·kg^−1^ by intragastric administration, *n* = 10). These doses adhere to the guidelines stipulated by the Chinese Pharmacopoeia, which specify a clinical dosage of 6–12 g per day. The Pharmacopoeia also suggests that with a doctor’s guidance, the maximum effective dose can be twice the usual maximum dose. As such, the dosages used in our study align strictly with these stipulations and mimic the therapeutic dosing applied in clinical practice. We also strictly followed these principles when administering domperidone. All our drug doses adhere to the guiding principle of “from the clinic, to the clinic”. The NC rats (*n* = 20) and FD model rats (*n* = 20) were given the same volume of normal saline. The drugs were administered continuously for 28 days, during which normal feeding was conducted. The body weight of each rat was recorded daily for subsequent evaluation. Ten 8-week-old rats each from the NC and FD model groups were randomly selected for the gastric emptying experiment, which was conducted according to the literature (Liu et al., 2008). At the end of the experiment and after 24 h of fasting, the rats were anesthetized with 5% chloral hydrate, and serum samples and stomach tissues were harvested. The full thickness of the stomach tissue, including the stomach body and the stomach wall, was stained with hematoxylin and eosin (H&E). The other tissue samples were frozen at −80°C for further analysis, whereas the serum samples were frozen at −20°C for analysis. All animal protocols were approved by the Ethics Committee of Jiangxi Provincial People’s Hospital (no. LLB20200518033) and were performed following the institution’s guidelines for the care and use of experimental animals.

### 2.14 ELISA

ELISA kits for interleukin 6 (IL-6), interleukin 1β (IL-1β), motilin (MTL), gastrin (GAS), acetylcholine (ACh) and acetylcholinesterase (AchE) supplied by Elabscience Biotechnology Co., Ltd. (Wuhan, China) were used to detect the content of the corresponding protein.

### 2.15 qRT‒PCR analysis

Total RNA was extracted using TRIzol® Reagent (Thermo Scientific, Waltham, MA, United States) and reverse-transcribed with oligo-DT using HiScript^TM^ Reverse Transcriptase (Vazyme, Beijing, China) according to the manufacturer’s instructions. The primers for PTGS1, PTGS2, DRD2, stem cell factor (SCF) and c-kit were synthesized by Tsingke (Beijing, China). GAPDH was used as an internal reference gene. The 2^−ΔΔCT^ method was used to determine the relative expression of each gene. The primers used in this study are provided in Supplementary Table S1.

qRT‒PCR was performed using SYBR^TM^ Green Master Mix (Vazyme) with a QuantStudio 6 Flex system (Applied Biosystems, Foster City, CA, United States). The PCR cycling profile was as follows: one cycle of 50°C for 2 min and 95°C for 10 min followed by 40 cycles of 95°C for 15 s and 60°C for 60 s. Fluorescence signals were detected using the QuantStudio 6 Flex system. Gene expression was normalized to that of the endogenous control GAPDH. The 2^−ΔΔCT^ method was used to determine the relative expression of each gene.

### 2.16 Western blotting analysis

According to our previously described method (Wang et al., 2020b), the stomach tissue protein levels of DRD2 were determined by Western blotting. GAPDH was used as the internal control. DRD2 and GAPDH antibodies were obtained from Affinity Biosciences Pty Ltd. (Affinity, Melbourne, Australia). Their corresponding secondary antibodies and protein markers were supplied by Boster Biological Technology Co., Ltd. (Boster, Wuhan, China).

### 2.17 Statistical analysis

All samples were qualitatively analyzed by UPLC–Q–TOF–MS/MS, and the raw data were processed using MarkerView v1.2.1 software (AB SCIEX). The data are presented as the means ± standard deviations (SDs). The significance of the results was determined by one-way analysis of variance using Prism 8.0.1 (GraphPad, San Diego, CA, United States). Tukey’s test was used for multiple comparisons if the variance was uniform, whereas the Tamhane test was used if the variance was not uniform. *p* < 0.05 was considered to indicate statistical significance.

## 3 Results

### 3.1 Qualitative analysis of the samples by UPLC–Q–TOF–MS/MS

The samples are shown in Supplementary Figure S1. The results from the UPLC–Q–TOF–MS/MS method validation are shown in Supplementary Table S2. Regarding precision, the RSDs of RT and peak area were 0.51% and 1.78%, respectively. The method repeatability yielded RSDs of the RT and peak area that were less than 2.13% and 4.59%, respectively. Moreover, the RSDs of the RT and peak area in terms of sample stability were less than 1.07% and 4.35%, respectively. These validation results suggested that the system precision, method repeatability, and sample stability were all in agreement with the requirements of qualitative analysis (RSD < 15%) (Fan et al., 2022).

**FIGURE 1:**
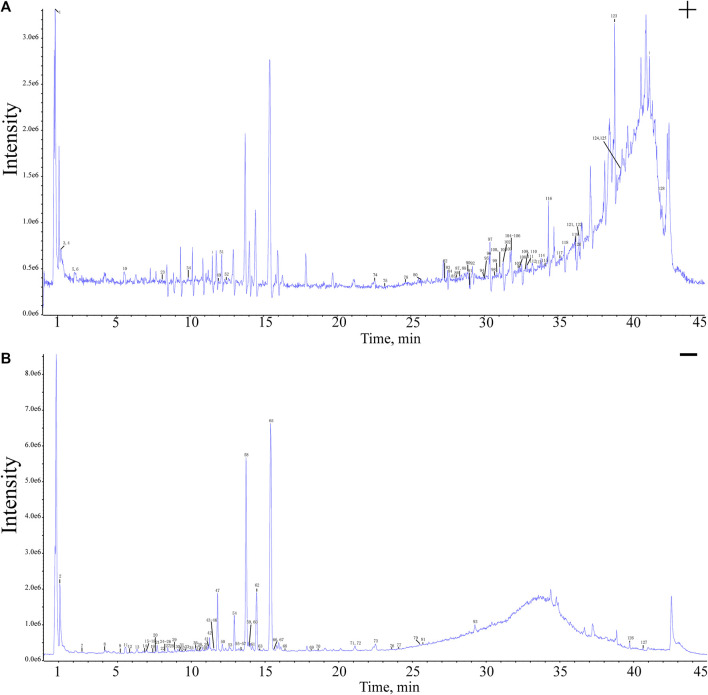
UPLC–Q–TOF–MS/MS TICs of the PAMR sample in the positive **(A)** and negative ion modes **(B)**.

To identify the metabolites of RAMR, raw AFI (RAFI), and PAMR, a combination of various methods was utilized for chemical analysis. First, the most reliable method was the identification of the target metabolites by comparison with the standards. In addition, the mass fragment ions (MS/MS ions) were analyzed to obtain the typical fragmentation pattern, and the structure was inferred based on the available literature data. Overall, a total of 128 metabolites were preliminarily identified from the analyzed samples. PAMR contained 127 of these metabolites, as shown in Figure 1. The details of the identified metabolites are shown in Supplementary Table S3. The asterisks in the table indicate that the metabolite was compared with a standard. Representative total ion chromatograms (TICs) of the standard metabolites and samples in the positive and negative ion modes are presented in Supplementary Figure S2. Supplementary Figure S3 shows the mass spectral properties of the standard metabolites in the positive and negative ion modes. As shown in the table, after processing with the RAFI decoction, the PAMR sample showed the presence of 5-hydroxymethyl furfural (5-HMF), which was not detected in RAMR. Moreover, unlike RAFI, PAMR included quercetin-7-O-rutinoside, whereas the content of 5,7,3′-trihydroxy-6,4′,5′-trimethoxyflavone was greatly reduced and below the detection limit. As the main metabolites of AMR, many lactones are considered the key differential marker metabolites, including atractylenolides and their isomers, especially the atractylenolide Ⅰ isomer.

### 3.2 Quantitative analysis of eight sample metabolites

Based on our previous studies (Wang et al., 2018; Yang et al., 2020), we determined the contents of 8 potentially active metabolites in this study (including synephrine, atractylenolide Ⅰ, atractylenolide II, atractylenolide III, atractylone, neohesperidin, hesperidin and naringin) by HPLC to investigate the influence of PAMR processing. The established quantitative HPLC method was validated based on its linearity, intraday and interday precision, repeatability, stability, recovery, limit of detection (LOD) and limit of quantitation (LOQ), as shown in Supplementary Table S4. Thus, the results revealed that the established method was precise for quantitative determination of the 8 selected metabolites.

**FIGURE 2:**
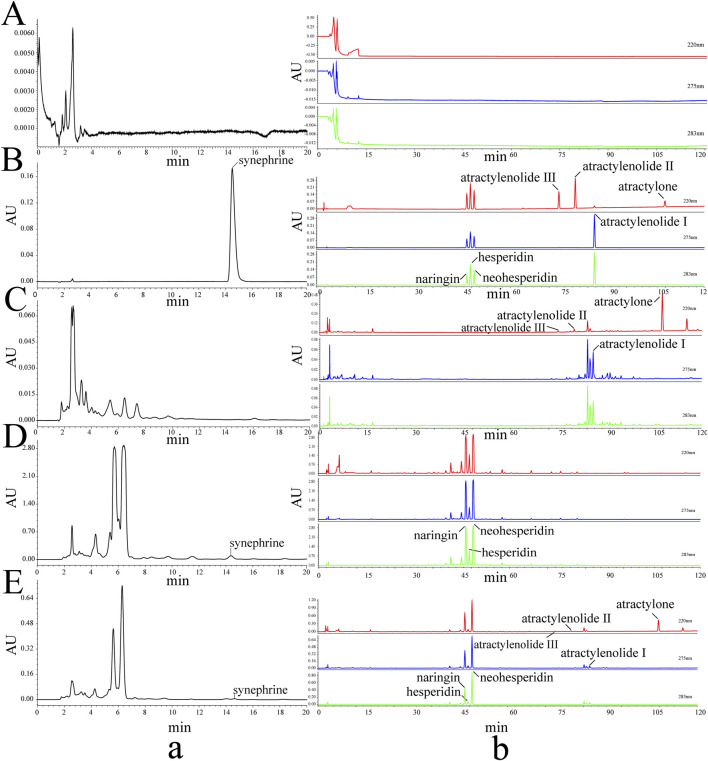
HPLC chromatograms of the 8 metabolites and different samples. Blank **(A)**, standards **(B)**, RAMR **(C)**, RAFI **(D)**, PAMR **(E)**, synephrine **(a)**, and the 7 other metabolites **(b)**.

To understand the changes in the 8 metabolites in the different sample groups that were present at different amounts, the samples were analyzed using validated HPLC analysis methods. The HPLC chromatograms are shown in Figure 2. The concentrations of the 8 metabolites were recorded, and their contents were calculated and are shown in Table 1.

**TABLE 1:** Contents of the 8 metabolites in the different samples. The results are shown as the means±standard deviations (SDs) (*n* = 3) (ND indicates that the metabolite was not detected).

Metabolite	Sample (mg·g^-1^) (*n* = 3)		
	RAMR	RAFI	PAMR
Synephrine	ND	6.1654 ± 0.3844	0.2739 ± 0.0126
Naringin	ND	41.1915 ± 2.8751	4.6897 ± 0.1588
Hesperidin	ND	7.5630 ± 0.3809	0.5227 ± 0.0180
Neohesperidin	ND	36.6905 ± 3.1014	3.6836 ± 0.1839
Atractylenolide Ⅰ	0.1649 ± 0.0065	ND	0.1529 ± 0.0091
Atractylenolide II	0.1441 ± 0.0127	ND	0.1451 ± 0.0031
Atractylenolide III	0.1222 ± 0.0009	ND	0.1413 ± 0.0073
Atractylone	7.4798 ± 0.3199	ND	6.5496 ± 0.5787

PAMR, as the processed product of RAMR and RAFI, contained synephrine, naringin, hesperidin and neohesperidin at different amounts after processing. Moreover, these results implied that the ancient processing method has an inconsistent effect on the changes in the metabolites of AMR. Classical AMR processing methods, such as frying, could significantly reduce the content of atractylone and increase the contents of atractylenolides Ⅰ, II, and III due to the poor stability of atractylone, which is easily oxidized at room temperature and then converted into the abovementioned atractylenolides (Hao et al., 2007), and the decomposition of atractylone is accelerated during heat-mediated processing (Wen et al., 1999). Moreover, atractylenolide III is dehydrated and decomposes into atractylenolide II after heat treatment (Wen et al., 1997). However, the metabolites in PAMR were changed in a different manner. This ancient and special processing method had little effect on increasing the contents of atractylenolides II and III but slightly reduced the content of atractylenolide Ⅰ. Of course, this method has some similarities with the classical processing method; that is, it can reduce the content of atractylone, although the reduction was small. We therefore speculated that the method presented here can transform atractylone into isomers of these lactones, but further extraction and separation are needed.

### 3.3 Screening of potentially active metabolites

The UPLC–Q–TOF–MS/MS results were utilized as the database for identifying potentially active metabolites. The screening criteria of OB ≥ 15% and DL ≥ 0.10 resulted in the identification of 25 metabolites that met the criteria. Although 7 metabolites did not meet the criteria, they were still listed as potentially active metabolites due to their clear biological activities to ensure that subsequent research was comprehensive and objective. First, although its values were lower than the set thresholds, synephrine (OB = 7.90%, DL = 0.04) has been suggested by the Chinese Pharmacopoeia to be the key metabolite of AFI. This a natural product derived from citrus has a structure that is similar to that of adrenergic receptor agonists, acts on some adrenergic and serotonergic receptors (Rossato et al., 2011) and regulates gastric intestinal motility (Fang et al., 2009). Synephrine has good anti-inflammatory properties and can affect the STAT-6 signaling pathway to inhibit the expression of eotaxin-1 (Roh et al., 2014). Moreover, the other 6 metabolites that were identified are flavonoids with strong biological activities. For example, hesperidin (OB = 13.33%, DL = 0.67) can regulate GI motility in both directions through H1 histamine receptors (Fang et al., 2009). Moreover, hesperidin also exerts anti-inflammatory (Lorzadeh et al., 2019), antitumor, antioxidant and analgesic effects (Aggarwal et al., 2020). Considering these factors, the screening conditions were expanded to include these metabolites. As a result, a total of 32 potentially active metabolites were identified in PAMR, as shown in Supplementary Table S5.

### 3.4 Network establishment and analysis

**FIGURE 3:**
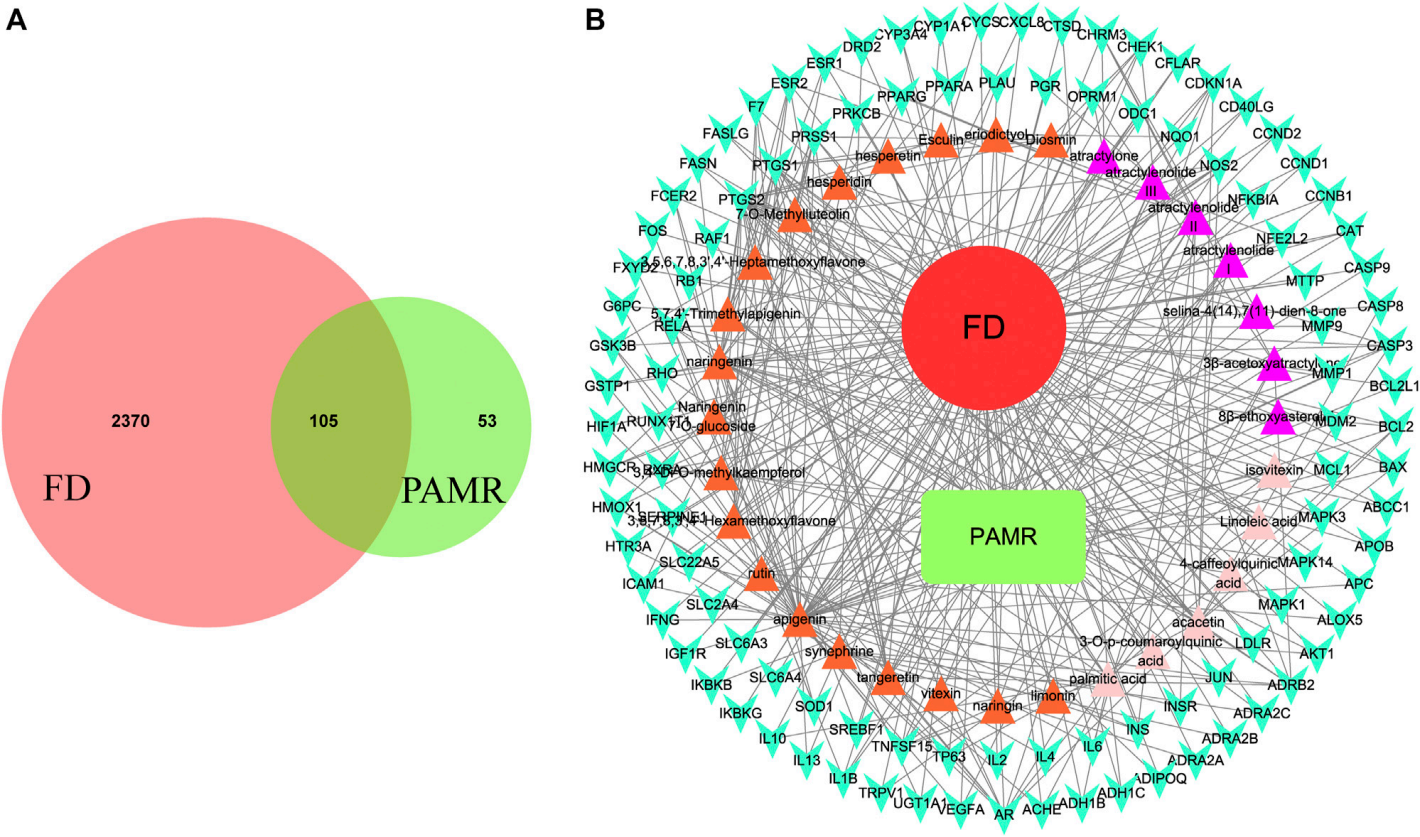
Results of the overlapping key targets. Overlapping key targets between PAMR and FD **(A)**. BMTD network **(B)**. The green node represents PAMR, and the red node represents FD. The 32 triangle nodes represent the potentially active metabolites in PAMR. The purple color indicates that the metabolite represented by the node was identified in AMR. The orange indicates that the node represents a metabolite found in AFI. The pink indicates that the metabolite is present in both AMR and AFI. The cyan V-shaped nodes represent the 158 overlapping key targets between FD and PAMR. The edges indicate that the linked nodes can interact with each other.

**FIGURE 4:**
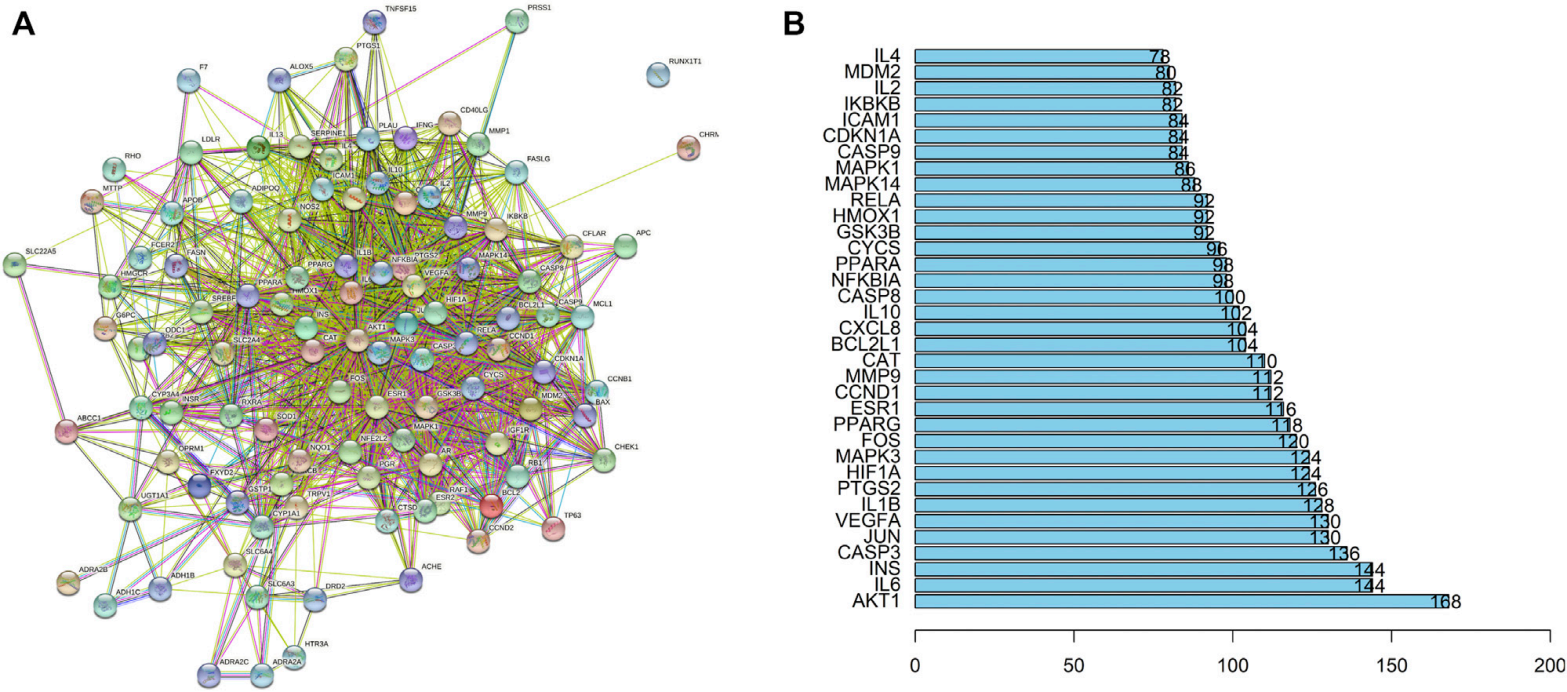
Results of the PPI analyses. PPI network **(A)**. Bar plot of the protein–protein interaction network **(B)**. The *X*-axis indicates the number of interacting proteins in the PPI network. The *Y*-axis indicates the target protein.

**FIGURE 5:**
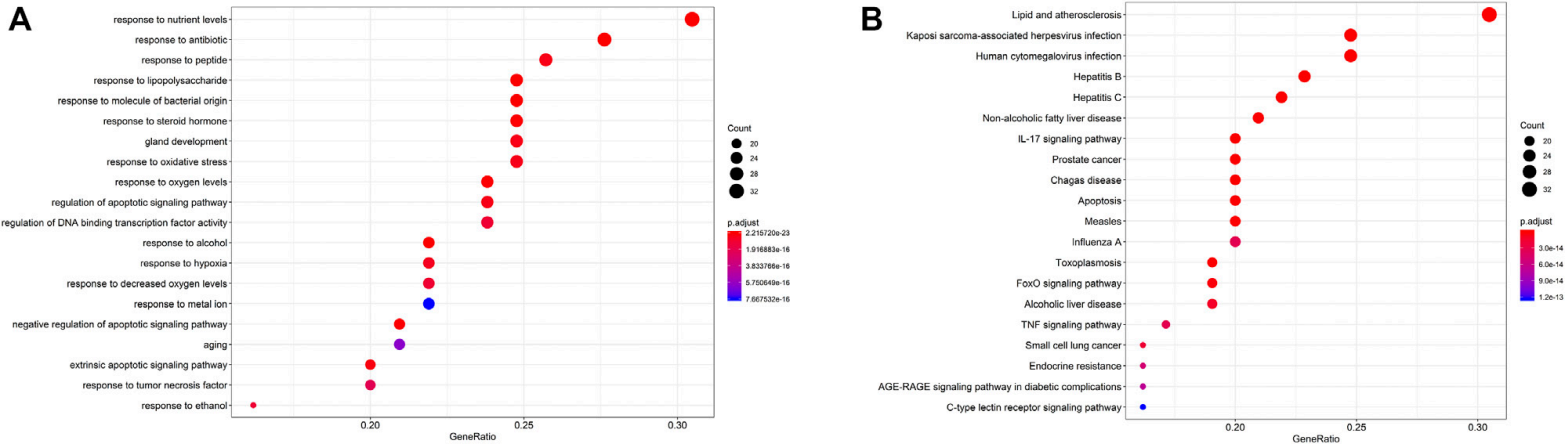
GO and KEGG pathway analyses. GO analyses of the 158 key targets associated with FD **(A)**. KEGG pathway enrichment analyses **(B)**. The *X*-axis indicates the number of targets enriched in the pathway. A redder color indicates a smaller *p*.adjust value as well as increased reliability and importance. A bluer color indicates a greater *p*.adjust value.

Among the 32 potentially active metabolites identified in PAMR, a total of 158 proteins were identified as their respective targets. Detailed information is provided in Supplementary Table S6. Additionally, a total of 2,452 targets related to FD were collected from the GeneCards database, and 24 related targets were obtained from the OMIM database. The targets were collated, and after removing duplicates, the targets were normalized using the UniProt database (www.UniProt.org), which ultimately yielded 2,357 therapeutic targets for FD. To find potential associations between PAMR and FD, we analyzed related targets of PAMR and FD, and a total of 105 overlapping targets were identified among these metabolites, as illustrated in Figure 3A. This finding indicates that PAMR is likely to treat FD through these 105 targets, as shown in Supplementary Table S7. To explore the potential mechanism underlying the therapeutic effect of PAMR on FD, a BMTD network based on the 32 potentially active metabolites and 105 prominent targets was constructed and visualized using Cytoscape software, as shown in Figure 3B. To further explore the relationship between the targets and analyze the mechanism of action underlying the efficacy of PAMR in treating FD, a PPI network was established. The network consisted of 105 nodes and 1570 edges, as shown in Figures 4A, B (the top 35 proteins). To unravel the biological processes and potential signaling pathways associated with the treatment of FD by PAMR, we performed Gene Ontology (GO) and Kyoto Encyclopedia of Genes and Genomes (KEGG) pathway enrichment analyses. The top 20 GO terms and KEGG pathways based on the *p*.adjust values are presented in Figures 5A, B.

### 3.5 Animal experiment results

**FIGURE 6:**
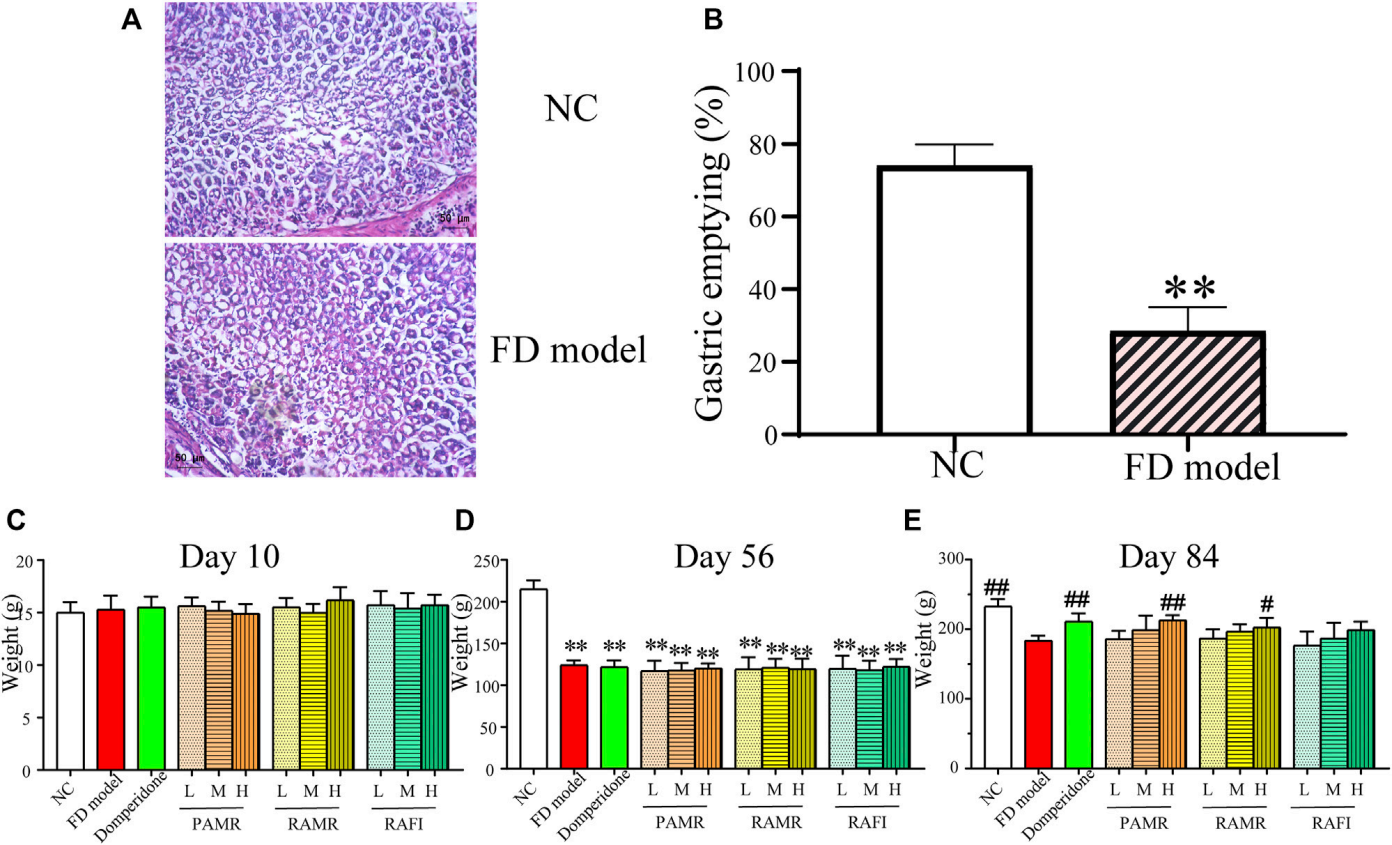
Evaluation of the FD model. H&E staining **(A)**. Results of the gastric emptying experiment **(B)**. Changes in the rat body weights on Day 10 just after the rat pups were gavaged with IAA and immediately before establishment of the FD model **(C)**. Day 56, completion of FD model establishment **(D)**. Day 84, completion of the 4 weeks of drug treatment **(E)**. *p < 0.05 and **p < 0.01 compared with the NC group; #*p* < 0.05 and ##*p* < 0.01 compared with the FD model group.

Eight-week-old rats in the normal control (NC) and FD model groups were randomly chosen for gastric tissue removal, and pathological sections were obtained. Section staining showed that the gastric mucosa morphology in the model group did not become abnormal compared with that in the NC group (Figure 6A). In the NC group, the gland arrangement was also normal, and no abnormalities were found. This result indicated that there were no organic lesions in the gastric tissue of the rats in the FD model group, which was consistent with the fact that FD is a nonorganic disease. The gastric emptying experiment with the 8-week-old rats showed that the gastric emptying rate of the rats in the FD model group was significantly lower than that of the rats in the NC group (*p* < 0.01), as displayed in Figure 6B. This finding demonstrates that the FD model rats exhibited significantly hindered gastric emptying compared with those in the NC group. Based on the above evaluation results and referring to the relevant literature (Liu et al., 2008; Wei et al., 2019), the FD model was successfully established. The body weights of the rats were recorded at each predetermined time point. The success of FD model establishment was verified by analyzing the recorded weight results. On Day 10, IAA intragastric administration was performed to create the FD model. At the end of modeling on Day 56 (week 8), the rats were treated, and treatment was completed on Day 84 (week 12). Before the administration of IAA for modeling (Day 10), no significant difference in body weight was found between the different groups (Figure 6C). After modeling was completed (Day 56), the body weights of the rats in the model group were significantly lower than those of the rats in the NC group (*p* < 0.01), indicating that the FD model was successfully established (Figure 6D). After treatment (Day 84), the body weights of the rats in the NC, domperidone, and PAMR-H groups were significantly higher than that of the rats in the FD model group (*p* < 0.01) (Figure 6E). Moreover, the body weight of the rats in the RAMR-H group were significantly higher than that of the rats in the FD model group (*p* < 0.05). The body weights of the rats in the other treatment groups were also higher than that of the rats in the FD model group, but the differences were not significant. However, a positive correlation between body weight and dosage was found in all the treatment groups. These results implied that domperidone, high-dose PAMR and high-dose RAMR can treat FD. Although no significant difference was detected, PAMR was slightly more effective than RAMR; thus, this result indicates that PAMR can treat FD more effectively than RAMR.

**FIGURE 7:**
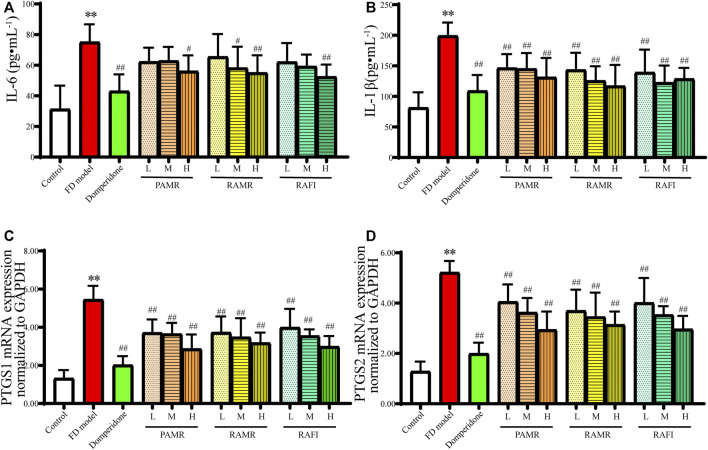
Anti-inflammatory effects. IL-1 **(A)** and IL-1β **(B)** concentrations in serum. mRNA expression of PTGS1 **(C)** and PTGS2 **(D)** in gastric tissue. **p < 0.01 compared with the NC group; ^#^ *p* < 0.05 and ^##^ *p* < 0.01 compared with the FD model group.

First, the anti-inflammatory effects of PAMR were verified. The results of the *in vivo* experiments showed that different doses of PAMR reduced the concentrations of IL-6 and IL-1β in the serum of FD model rats (Figures 7A, B). Different doses of each drug caused significant decreases in the mRNA expression levels of PTGS1 and PTGS2 (*p* < 0.01), as shown in Figures 7C, D. In addition, these anti-inflammatory effects were dose dependent. Notably, the results also showed that RAMR and RAFI had clear anti-inflammatory effects when used alone. However, the anti-inflammatory effect of PAMR was slightly greater than those of RAMR and RAFI, although the difference was not significant. These results also prove the results from the network analysis. Therefore, it can be concluded that multiple potentially active metabolites of both AMR and AFI can regulate these inflammatory targets, indicating that the anti-inflammatory effect of RAMR is enhanced after processing.

**FIGURE 8:**
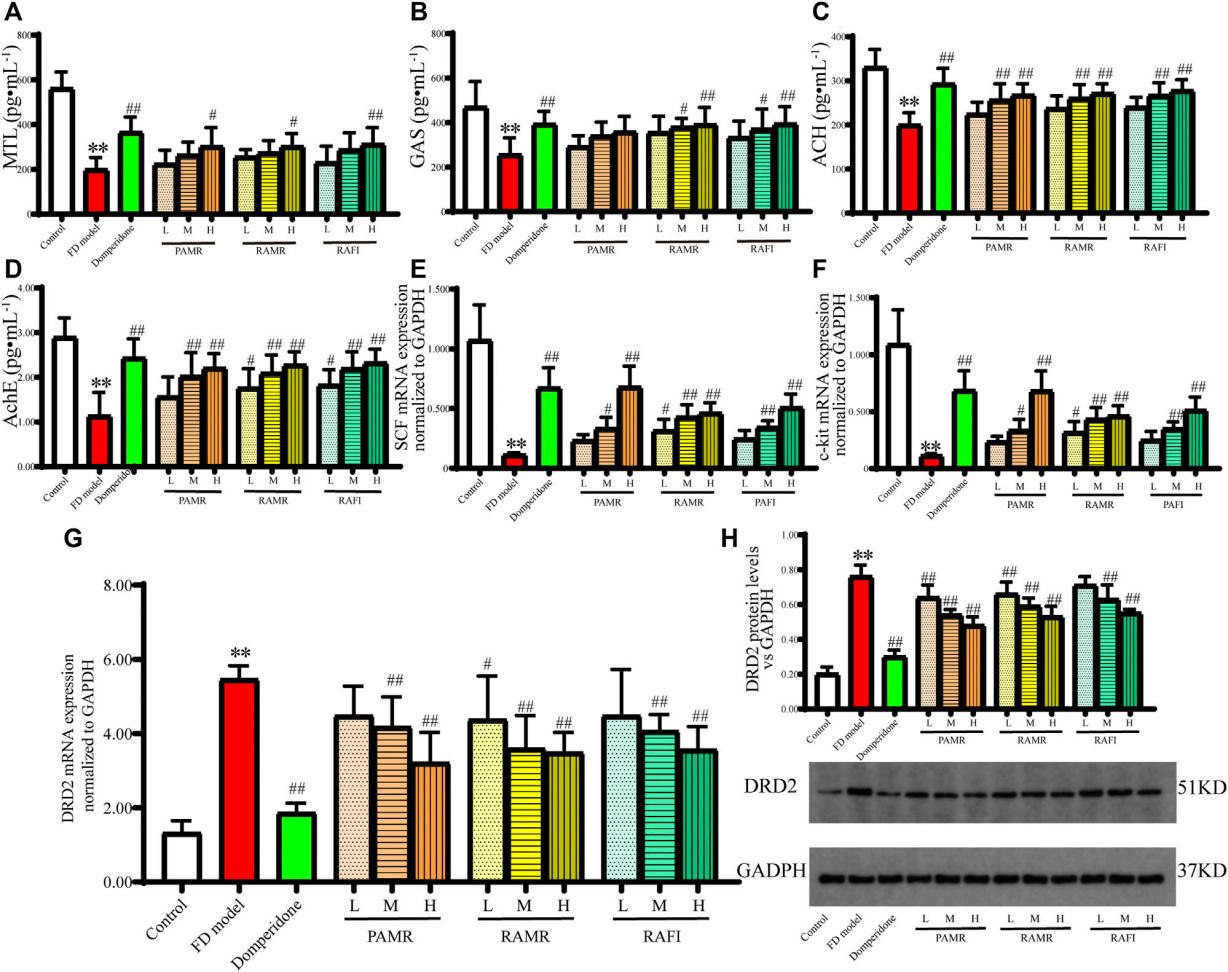
Gastric motility effects. MTL **(A)**, GAS **(B)**, Ach **(C)** and AchE **(D)** concentrations in serum. mRNA expression of SCF **(E)**, c-kit **(F)** and DRD2 **(G)** in gastric tissue. Protein expression of DRD2 in gastric tissue **(H)**. **p < 0.01 compared with the NC group; ^#^ *p* < 0.05 and ^##^ *p* < 0.01 compared with the FD model group.

Further exploration was conducted to examine the effects of PAMR on gastric motility in the context of treating FD (Figure 8). MTL is thought to be a unique hormone capable of acting during the digestive interphase rather than postprandially (Ohno et al., 2010). Many studies have shown that the motilin receptor exists in smooth muscle cells (Strunz et al., 1975; Adachi et al., 1981), where it exerts its direct contractile effect. GAS is secreted by G cells in the gastric antrum, functions as an acidic secretor and regulates various biological processes as a cell growth factor (Dockray et al., 2001). Therefore, MTL and GAS are considered important indicators of GI motility. As shown in Figures 8A, B, the MTL concentrations in the serum of rats in the domperidone group and the groups receiving high dosages of various drugs were significantly higher than those in the serum of rats in the FD model group (*p* < 0.01). Similarly, the concentrations of GAS in the serum of rats in the domperidone, RAMR high-dose and RAFI high-dose groups were significantly higher than those found in the FD model group (*p* < 0.01). However, the concentration of GAS in the PAMR group was higher than that in the FD model group, but the increase was not significant (*p* > 0.05). Additionally, PAMR did not regulate the MTL and GAS concentrations as effectively as RAMR and RAFI, but no significant differences were observed (*p* > 0.05).

The concentrations of Ach and AchE in the serum of each treatment group increased following drug administration (Figures 8C, D). However, the regulation by PAMR was still weaker than that by RAMR and RAFI, although no significant difference was found (*p* > 0.05). Ach can induce muscle contraction and promote intestinal peristalsis and gland secretions, ultimately accelerating intestinal emptying. However, the mechanism of PAMR is different from that of traditional AchE inhibitors, which improve GI motility by inhibiting AchE secretion and increasing Ach secretion. The hypothesis suggests that PAMR may directly enhance the secretion of Ach and thereby promote GI motility. The increased Ach secretion also increases the secretion of AchE. Nonetheless, further verification is needed to explain this mechanism.

Modern research has found that interstitial cells of Cajal (ICCs) with electrical rhythmicity can produce slow waves and electrical excitation conduction (Langton et al., 1989). ICCs also form a network with the GI nervous system and GI smooth muscle cells, which is known as the ENS–ICC–SMC network. This network is distributed throughout the GI system and can regulate GI motility. Modern medical studies have proposed that ICCs are closely related to GI motility disorder (Yin and Chen, 2008), which is one of the most important causes of FD. C-kit is specifically expressed by ICCs and can regulate the proliferation and differentiation processes of ICCs when combined with its specific ligand SCF (Lin et al., 2016; Zhang et al., 2016). The mRNA expression levels of SCF and c-kit in the gastric tissue of rats were increased by the middle and high doses of each drug (Figures 8E, F). The mRNA expression levels of SCF and c-kit in the PAMR-H group were higher than those in the RAMR and RAFI groups and similar to those in the domperidone group.

High DRD2 expression could lead to the colon being underpowered and the generation of movement disorders. The mRNA and protein expression levels of DRD2 in the gastric tissue of rats in the FD model group were significantly higher than those found in the NC group (*p* < 0.01), as shown in Figures 8G, H. The mRNA expression levels of DRD2 in all treatment groups, with the exception of the RAMR-L group, were significantly decreased (*p* < 0.01) in a dose-dependent manner compared with those in the FD model group. However, PAMR regulated the DRD2 mRNA levels slightly better than RAMR and RAFI, but the difference was not significant (*p* > 0.05). The analysis of DRD2 protein expression supported this finding.

The results from this series of analyses suggests that the processing mechanism of PAMR primarily enhances the regulation of certain targets associated with promoting gastric motility rather than enhancing the regulation of all related targets. Specifically, the processing of PAMR can weaken the regulation of MTL, GAS, Ach, and AchE and enhance the impact on DRD2, SCF, and c-kit.

## 4 Discussion

Traditional technologies for the processing of natural botanical drugs under the theory of TCM are worthy of protection and research. In this study, PAMR was prepared following a processing method documented in ancient books. Additionally, a network analysis-based strategy adopting a variety of modern research techniques was utilized, and the mechanism of the combination method was expounded from the aspects of chemical composition, pharmacodynamics and pharmacology. Moreover, the therapeutic role of the aqueous extracts of PAMR in the treatment of FD was elucidated. Our qualitative analysis indicated the generation of 2 new metabolites after processing. The first is 5-HMF, a furfural metabolite containing a furan ring structure that is formed by the dehydration of glucose, fructose and other monosaccharides or six-carbon sugar compounds at high temperature, caramelization or the Maillard reaction. 5-HMF is a classical product that is among the commonly produced compounds (Van Putten et al., 2013) in Chinese medicine and is generated or produced at much larger amounts after processing (Wang et al., 2020a). However, the practical applications of 5-HMF are controversial. Injections containing 5-HMF can cause adverse clinical reactions, such as anaphylactic shock and respiratory system damage (Jin and Cai, 2009; Sun and Tang, 2016). However, a low dose of 5-HMF has biological activities, such as antioxidant, antitumor, learning and memory improvement, and antiallergic effects (Yalcin and Cabrales, 2012; Zhao et al., 2013; Li et al., 2015). In this study, the presence of 5-HMF was not detected in RAMR or RAFI but was detected in PAMR. This phenomenon is believed to align with the conditions of the processing reaction. The reaction was positively correlated with temperature to a certain extent, and the moisture content had a great influence. In an environment with little or no water content, the reaction is very difficult to conduct. However, the environmental conditions during PAMR processing were exactly those required by the Maillard reaction. Therefore, it is believed that 5-HMF could serve as an indicator to assess the degree of processing but may not be suitable for establishing quality standards. The other newly generated compound was quercetin-7-O-rutinoside, which has the ability to scavenge DPPH free radicals and thus exhibits significant antioxidant activity (Cao et al., 2013). Additionally, its bacteriostatic activity was found to be comparable to that of a standard antibiotic, amoxicillin equivalent (*p* > 0.05), which indicates that quercetin-7-O-rutinoside may regulate the intestinal flora *in vivo* by inhibiting the activity of pathogenic bacteria such as *Escherichia coli* and *Pseudomonas aeruginosa* to ultimately treat GI diseases (Jarial et al., 2018). Based on the combined qualitative and quantitative analysis results obtained in this study, it can be speculated that the observed changes in the effects of PAMR could be attributed to alterations in its metabolites and potentially associated with lactone isomers. However, further evidence is needed to support this hypothesis. Therefore, the intention of our next study is to develop methods for extracting and separating these isomers, which would allow more accurate identification and characterization of the compounds. These findings will contribute to a better understanding of the underlying principles of this ancient and distinctive processing method.

After establishing the various networks, a series of analyses were conducted to elucidate the underlying mechanism of PAMR in the treatment of FD. First, the roles of the nodes in the BMTD network were quantified to assess their significance. Two important parameters, degree (the number of edges connected to a node) and betweenness centrality (BC) (Brandes, 2001), were introduced for this purpose. BC measures the importance of nodes in the network and identifies the nodes that are more important (Grobelny et al., 2018). In general, degree and intermediate degree were positively correlated. Therefore, nodes with a relatively high BC value were considered important in the network, even if their degree value was not sufficiently high. We noticed that the BC of DRD2 (degree = 6) was 0.00469, which was higher than that of CDKN1A (BC = 0.00391) and RXRA (BC = 0.00459), both of which had the same degree; this finding implies that DRD2 was more worthy of our attention. Interestingly, DRD2 is a major GI receptor. Therefore, DRD2 antagonists such as domperidone could reduce dopamine-mediated gastric smooth muscle relaxation by blocking this target and thereby treat FD (Xiang et al., 2015). In this network, the average degree value for each metabolite was 8.3, and the average degree value for each target was 3.2. The results from the BMTD network analysis indicated that PAMR had multimetabolite multitarget characteristics for the treatment of FD, which is also a favorable illustration of the multimetabolite multitarget characteristics of TCM. Notably, the degrees of the flavonoids were higher. Among these metabolites, apigenin had the highest degree (51), followed by naringenin (24) and naringin (4). *In vivo* experiments have shown that apigenin can not only inhibit PTGS2 and PGE2 (Kiraly et al., 2016) but also reduce the production of cytokines such as IL-1β, IL-6 and TNF-ɑ by inhibiting the PTGS2 and NF-κB pathways to achieve anti-inflammatory effects (Wang et al., 2014). Moreover, *in vivo* and *in vitro* experiments have suggested that naringin has not only anti-inflammatory and antioxidant activities (El-Desoky et al., 2018) but also antidiabetic properties in type 2 diabetic rats (Ahmed et al., 2017). Nevertheless, given the unique nature of the processing method employed in this study, the flavonoids derived from RAFI constituted only a minor fraction in PAMR. Therefore, our focus primarily centered on the metabolites originating from RAMR. The results of the BMTD network analysis showed that sesquiterpenes, such as atractylenolide Ⅰ (degree = 5), atractylenolide II (degree = 11), atractylenolide III (degree = 2) and atractylone (degree = 5), also had high degree values. In fact, some of their key biological activities in the treatment of FD have been experimentally verified (Zhang et al., 2019; Yan et al., 2020). In addition, atractylenolide II can simultaneously act on 6 targets, namely, PTGS1, PTGS2, CHRM3, DRD2, IL-6 and IL-1β, which is also an embodiment of the multiple targets of TCM. The results of this study demonstrated that the multimetabolite characteristics of PAMR for the treatment of FD may be first reflected in the 32 potentially active metabolites. This finding was further reflected by the fact that a single target can be simultaneously affected by multiple potentially active metabolites. For example, PTGS2 had the highest degree value among the targets (25), followed by PTGS1 (14). The findings from the BMTD network analysis indicate that sesquiterpenes present in AMR, including atractylenolide I and 8β-ethoxyasterolid, could potentially influence the activity of PTGS2. PTGS2 can also be regulated by flavonoids such as hesperetin, hesperidin and tangeretin in AFI. These results indicated that the same target can be regulated by multiple potentially active metabolites in PAMR, suggesting that these potentially active metabolites have potential synergistic effects in the treatment of FD. These data also explain the mechanism of AMR processing by AFI decoction to some extent; that is, these two Chinese medicines could produce synergistic effects to enhance the therapeutic effect of PAMR.

Based on the results from our network analyses, we speculated that PAMR exerts therapeutic effects on FD mainly through its anti-inflammatory and gastric motility activities. The first property to be investigated was the anti-inflammatory effect of PAMR. FD is always accompanied by inflammation, which is an important factor. In the BMTD network, two intriguing proteins, PTGS1 and PTGS2, were identified. These proteins belong to different isoforms of cyclooxygenase and play crucial roles in the conversion of arachidonic acid into prostaglandins. PTGS1 and PTGS2 are similar to a certain extent but are still different, similar to twins. Due to their similar biological activities, these proteins can both promote the production of PGE_2_ (Caughey et al., 2001; Chambers et al., 2020). Therefore, PTGS1 and PTGS2 are the targets of aspirin and other nonsteroidal drugs, which can exert anti-inflammatory, antipyretic and analgesic effects through inhibition of these factors (Vane, 1971). The top 20 biological processes included targets closely related to inflammation, such as PTGS1, PTGS2, IL-1β, IL-6, and IL-10. Moreover, the KEGG enrichment analysis results displayed many signaling pathways that could directly or indirectly participate in the inflammatory response. These results strongly suggest the importance of considering the role of PAMR in inflammation when treating FD. Additionally, according to the constructed networks, we concluded that the treatment of FD by PAMR was also related to GI motility. For example, FD has been associated with delayed gastric emptying and receptive gastric relaxation (Sarnelli et al., 2003; Enck et al., 2017). Dopamine produces a concentration-dependent relaxation effect on the colon, and high dopamine concentrations eliminate spontaneous motility in isolated rat colons (Aguilar et al., 2005). DRD2 is a dopamine receptor widely distributed in the intestinal nervous system, such as the stomach, duodenum, ileum and colon. In DRD2-knockout mice, the total GI transport time is significantly reduced, indicating a significant increase in GI motility (Li et al., 2006). Therefore, DRD2 is an effective drug target for promoting GI motility. The analysis of the PPI network identified the target AchE, which hydrolyzes Ach at a high rate to reduce its content (Quinn, 1987). As a result, the smooth muscle of the GI tract cannot be excited to produce strong contractions (Sims et al., 1997). Therefore, AchE was identified as a highly effective target. Moreover, our analysis showed that the mechanism by which PAMR treats FD is very complex and extremely diverse, which is consistent with the multimetabolite and multitarget characteristics of TCM and its concept of holistic treatment.

The analysis of the BMTD network established in this study predicted that isovitexin, a metabolite found in both AMR and AFI, could regulate inflammation by influencing NF-κB. This prediction is consistent with previous substantial confirmations of such an effect (Lv et al., 2016). Indeed, the presence of cytokines from the interleukin family, including the proinflammatory cytokines IL-1β, IL-2, and IL-6 and the anti-inflammatory cytokines IL-4, IL-10, and IL-13, was identified in the PPI network analysis. In a previous study, we found that atractylenolide Ⅰ has a significant anti-inflammatory effect *in vitro* by dose-dependently reducing IL-1β and IL-6 produced by lipopolysaccharide (LPS)-stimulated RAW 264.7 macrophages (Yang et al., 2020). GI motility disorder caused by GI inadequacy is a common cause of FD. In fact, the most widely used GI motility drugs, such as domperidone and metoclopramide, are DRD2 antagonists. Although the degree of DRD2 in the PPI network was not very prominent, DRD2 could interact with synephrine, atractylenolides Ⅰ, II, and III and atractylone in the BMTD network. Thus, PAMR has excellent potential to improve GI motility by targeting DRD2. AchE is a highly effective target, and AchE inhibitors such as neostigmine and huperzine A have been shown to inhibit AchE and improve GI motility (Zhang et al., 2013b; Parthasarathy et al., 2015). Paradoxically, several studies have shown that AMR can promote GI motility while increasing the AchE content in animals with gastric motility deficiency (Zhu et al., 2003; Liu et al., 2009). Unfortunately, these studies did not measure the Ach contents in the animals. This unusual phenomenon puzzled us to a great extent. However, in TCM theory and clinical practice, AMR can be used to treat constipation (Zhou et al., 2019) and diarrhea (Ma et al., 2020). Based on these considerations, it can be hypothesized that AMR has a bidirectional regulatory function in the treatment of FD. However, the specific mechanism of intervention targeting AchE and GI motility remains to be explored. Therefore, this study aimed to conduct experimental verification to preliminarily explore this issue. Therefore, our suspicion is that AMR might increase the secretion of choline acetyltransferase (ChAT) or Ach, leading to a reverse stimulation of AchE production and thereby promoting GI motility. Additionally, the presence of steroid receptors ESR1, ESR2, and AR in the PPI network caught our interest because these receptors are closely associated with sex hormones. Statistical analyses have shown that women are more likely to suffer from FD than men (Olafsdottir et al., 2010; Napthali et al., 2016). The presence of the steroid receptors ESR1, ESR2, and AR in the PPI network is a fascinating phenomenon. Based on relevant modern studies (Schipper, 2016), this finding may be associated with the higher susceptibility of females to endocrine disorders related to sex hormones. Estrogen has been shown to play an anti-inflammatory role in the GI tract by modulating numerous proteins involved in immune system regulation (Straub, 2007). Both estrogen and androgens regulate colonic motility, suggesting a role for sex hormones in GI diseases (Hutson et al., 1989). Moreover, one study showed that preconditioning the distal colon of male rats with testosterone can enhance spontaneous contraction of the colon (Baumgartner et al., 2017). Therefore, ESR1, ESR2 and AR might be effective targets in the pathogenesis of FD. Based on the aforementioned rationale, the following hypothesis can be proposed: PAMR can potentially alleviate GI disorders by modulating sex hormones and their associated targets within the GI system. In summary, the main therapeutic focus of PAMR is directed toward addressing inflammation and GI dyskinesia, which are two underlying causes of FD. PAMR exhibits anti-inflammatory properties and mediates GI motility to effectively treat FD. The experimental verification results showed little difference among the relevant indicators of PAMR, RAMR and RAFI. Although our current sample size may not be sufficient for a comprehensive study, emerging trends suggest that the significance of these findings may improve with a larger sample size. While there may still be a gap due to the limitations of our current sample size, our future studies will aim to address this issue by further increasing the sample size.

Although AFI and especially AMR have been used in TCM to treat GI diseases, there have been a few reports of GI side effects. Thus, the safety of these botanical drugs remained the focus of our attention. We noticed that at concentrations greater than 200 μg·mL^−1^, naringin exhibited cytotoxicity to human bone marrow mesenchymal stem cells (Zhang et al., 2009). Thus, we strictly controlled the dose of RAFI used during PAMR processing, and this dose was one-tenth the weight of the main drug, RAMR. Therefore, at this dose, it is believed that naringin in PAMR would have limited potential for inducing side effects while still providing therapeutic benefits. The results also showed that although the significant proteins PTGS1 and PTGS2 share many similarities, a notable disparity in their expression levels was observed. PTGS1 is constitutively expressed in a wide range of cells and tissues, and its expression remains largely unchanged in most diseases. Therefore, PTGS1 was the main source of PGE_2_ in normal tissues. In general, PTGS2 is present at undetectable levels in most normal cells and tissues and is only expressed in inflammatory cells (Vane et al., 1998). Moreover, the prostaglandins synthesized by PTGS1 and PTGS2 protect the stomach and intestines (Gudis and Sakamoto, 2005). NSAIDs such as aspirin can cause serious GI side effects because they inhibit both cyclooxygenase and the prostaglandins that protect the gastric mucosa (Vane and Botting, 2003). Therefore, our next objective was to investigate the underlying reasons for the low or nontoxic nature of natural botanical drugs that can be consumed as functional foods, such as AMR.

## 5 Conclusion

To clarify the principle of the AMR herbal processing method used in this study, which has been documented in ancient books, the study employed a network analysis strategy. Qualitative and quantitative analyses were performed using UPLC-Q-TOF-MS/MS and HPLC techniques. A total of 127 metabolites were identified in PAMR. Unlike the metabolites found in unprocessed RAMR and RAFI, 5-HMF and quercetin-7-O-rutinoside were newly formed in the product of PAMR processing, and the level of 5,7,3′-trihydroxy-6,4′,5′-trimethoxyflavone decreased to below the detection limit after processing. Quantitative studies showed that the contents of 8 major metabolites in PAMR changed significantly after processing compared with their levels before processing. Subsequently, various networks, including the BMTD network and PPI network, were established for predictive analysis. The findings suggest that the mechanism of action underlying the efficacy of PAMR in treating FD can be primarily attributed to its anti-inflammatory effects and promotion of gastric motility. These preliminary results were confirmed in subsequent *in vivo* experiments. The enhanced aqueous extracts of processed AMR (PAMR) demonstrate improved efficacy in treating FD, indicating that this pharmaceutical technology enhances the anti-inflammatory trend and promotes gastric motility by modulating DRD2, SCF, and c-kit. However, this enhancement comes at the cost of attenuating the regulation of MTL, GAS, Ach, and AchE. Through this series of investigations, we aimed to unravel the factors underlying the efficacy of this herbal formulation in improving FD in clinical settings. The aim of this series of explorations was to better protect and preserve this ancient, mysterious, and interesting herbal processing method.

## Data Availability

The original contributions presented in the study are included in the article/Supplementary Material, further inquiries can be directed to the corresponding authors.

## Abbreviations

AFI, Aurantii Fructus Immaturus; AMR, Rhizoma Atractylodis Macrocephalae; BMTD, botanical drug–metabolite–target-disease; DL, drug likeness; FD, functional dyspepsia; GO, Gene Ontology; IL, interleukin; KEGG, Kyoto Encyclopedia of Genes and Genomes; OB, oral bioavailability; OMIM, Online Mendelian Inheritance in Man; PAMR, Rhizoma Atractylodis Macrocephalae processed by Aurantii Fructus Immaturus decoction; PPI, protein–protein interaction; PTGS1, prostaglandin G/H synthase 1; PTGS2, prostaglandin G/H synthase 2; RAFI, raw Aurantii Fructus Immaturus; RAMR, raw Rhizoma Atractylodis Macrocephalae; STRING, Search Tool for the Retrieval of Interacting Genes/Proteins; TCM, traditional Chinese medicine; 5-HMF, 5-hydroxymethyl furfural.
