# Supplementary material for: Rhizoma Atractylodis Macrocephalae—Assessing the influence of herbal processing methods and improved effects on functional dyspepsia
Source: Front Pharmacol. 2023 Aug 4;14:1236656. doi: 10.3389/fphar.2023.1236656 (PMC10436233; doi:10.3389/fphar.2023.1236656)
Supplement: Supplementary file 1 [file Table1.DOCX]

Unveiling the Influence of Herbal Processing Method on Rhizoma Atractylodis Macrocephalae Compounds and Enhancing the Improvement of Functional Dyspepsia

Song-Hong Yang ^1^§, Jing Zhu ^1^§, Wen-Ting Wu ^1^§, Jun-Mao Li ^1^, Heng-Li Tong ^1^, Yi Huang ^1^, Qian-Feng Gong ^1^, Fei-Peng Gong ^1,2^*, Ling-Yun Zhong ^1^*

^1^School of Pharmacy, Jiangxi University of Chinese Medicine, Nanchang 330004, China

^2^Jiangxi Provincial People's Hospital, The First Affiliated Hospital of Nanchang Medical College, Nanchang 330006, China

**§These authors contributed equally to this work and share first authorship**

*** Correspondence:**Fei-Peng Gong: 13027211861@163.com; Ling-Yun Zhong: ly1638163@163.com

Table S1 The primer sequences uesd for qRT-PCR analysis

| Name | Primer | Sequence | Size |
| --- | --- | --- | --- |
| GAPDH | Forward | 5‘- ACAGCAACAGGGTGGTGGAC -3’ | 253bp |
|  | Reverse | 5‘- TTTGAGGGTGCAGCGAACTT -3’ |  |
| PTGS1 | Forward | 5‘- ACCCACCTTCCGTAGAACAG -3’ | 166bp |
|  | Reverse | 5‘- TTCAGCAAGTCACACACACG -3’ |  |
| PTGS2 | Forward | 5‘- AGAGCAGAGAGATGAAATACC -3’ | 266bp |
|  | Reverse | 5‘- AGGAGAACAGATGGGATTAC -3’ |  |
| DRD2 | Forward | 5‘- GCAGACAGGCCCCACTACAACT -3’ | 194bp |
|  | Reverse | 5‘- TAGACAACCCACGGCATTACCA -3’ |  |
| SCF | Forward | 5‘- CAAAACCATTTATGTTACCCCCT -3’ | 283bp |
|  | Reverse | 5‘- TACACCTCTTGAAACTCTCTCTC -3’ |  |
| C-kit | Forward | 5‘- TCTGGTCCTACGGCATCTTC -3’ | 239bp |
|  | Reverse | 5‘- GCTCTCTGAGATCTGCGTCT -3’ |  |

Table S2 The results of the precision, repeatability and stability of the UPLC-Q-TOF-MS/MS (n=6)

|  | RSD of RT | RSD of peak area |
| --- | --- | --- |
| precision | 0.51% | 1.78% |
| repeatability | 2.13% | 4.59% |
| stability | 1.07% | 4.35% |

Table S3 Detailed information on the 128 identified compounds.

| No. | t_R_ (min) | Formula | Positive/Negative ion ([M+H]^+^/[M-H]^-^ m/z) | | | | Identification | Intensity/Source | | |  |
| --- | --- | --- | --- | --- | --- | --- | --- | --- | --- | --- | --- |
|  |  |  | Theoretical  mass (Da) | Measured  mass (Da) | Error (ppm) | MS/MS (m/z) |  | RAMR | RAFI | PAMR | |
| 1^*^ | 0.98 | C_9_H_13_NO_2_ | 168.1019 | 168.1020 | 0.6 | 150.0932(65%) [M+H]^+^, 135.0686(91%), 107.0503(100%), 91.0558(77%), 77.0399(39%) | Synephrine | ND | 5537 | 1563 | |
| 2 | 1.10 | C_6_H_8_O_7_ | 191.0197 | 191.0205 | 4.2 | 191.0199(9.1%) [M-H]^-^, 129.0202(9.1%), 111.0106(100%), 87.0126(74%), 85.0336(38%), 67.0218(22%), 57.0435(12%) | Citric acid | 24434 | 9408 | 8410 | |
| 3 | 1.20 | C_6_H_6_O_3_ | 127.0390 | 127.0393 | 2.9 | 127.0392(61%) [M+H]^+^, 109.0294(34%), 81.0365(100%), 69.0005(48%), 53.0463(33%) | 5-Hydroxymethyl furfural | ND | ND | 22122 | |
| 4 | 1.23 | C_7_H_6_O_2_ | 123.0441 | 123.0444 | 2.4 | 123.0914(6.1%) [M+H]^+^, 81.9401(76%), 77.0419(92%), 55.9395(100%) | Benzoic acid | 3565 | ND | 3565 | |
| 5 | 2.17 | C_9_H_11_NO_2_ | 166.0863 | 166.0859 | -1.9 | 120.0820(100%) [M+H]^+^, 103.0562(63%), 91.0563(13%), 77.0421(36%) | Dimethyl anthranilate | 5055 | 11266 | 10053 | |
| 6 | 2.17 | C_8_H_9_N | 120.0808 | 120.0814 | 4.8 | 120.0816(27%) [M+H]^+^, 103.0560(69%), 91.0564(39%), 77.0420(100%) | 2-Ethenylbenzenamine | 7750 | 48245 | 29543 | |
| 7 | 2.56 | C_13_H_16_O_9_ | 315.0722 | 315.0718 | -1.0 | 315.0720(74%) [M-H]^-^, 153.0199(48%), 152.0119(100%), 109.0341(22%), 108.0237(54%), 85.0335(6.3%) | 3-Hydroxybenzoic acid-4-O-glc | 138294 | 58873 | 90643 | |
| 8 | 4.09 | C_16_H_18_O_9_ | 353.0878 | 353.0868 | -2.7 | 353.0872(8.9%) [M-H]^-^, 191.0556(100%), 179.0339(47%), 135.0460(35%) | 3-Caffeoylquinic acid | 33276 | 13164 | 4698 | |
| 9 | 5.22 | C_15_H_16_O_9_ | 339.0722 | 339.0715 | -1.8 | 339.0715(2.3%) [M-H]^-^, 177.0206(100%), 133.0320(16%) | Aesculin | ND | 250 | 3370 | |
| 10 | 5.54 | C_21_H_36_O_10_ | 466.2647 | 466.2639 | -1.6 | 269.1758(21%) [M+NH_4_]^+^, 251.1641(100%), 233.1535(95%), 233.1686(13%), 215.1430(56%), 191.1421(28%) | Atractyloside A | 1874 | ND | 1056 | |
| 11 | 5.63 | C_16_H_18_O_8_ | 337.0929 | 337.0925 | -1.1 | 337.0924(14%) [M-H]^-^, 163.0403(100%), 119.0540(28%) | 3-O-p-Coumaroylquinic acid | 400 | 2933 | 350 | |
| 12 | 5.82 | C_15_H_16_O_9_ | 339.0722 | 339.0710 | -3.3 | 339.0707(5.4%) [M-H]^-^, 219.0329(5.4%), 201.0185(5.4%), 177.0196(100%), 133.0321(5.4%), 109.0356(5.4%) | Cichoriin | ND | 2193 | 2800 | |
| 13 | 6.33 | C_16_H_18_O_9_ | 353.0878 | 353.0866 | -3.4 | 353.0866(1.8%) [M-H]^-^, 191.0564(100%) | 5-Caffeoylquinic acid | 215258 | 3322 | 12632 | |
| 14 | 6.88 | C_9_H_8_O_4_ | 179.0350 | 179.0358 | 4.4 | 179.0288(13%) [M-H]^-^, 135.0465(100%), 107.0501(6.5%) | Caffeic acid | 3863 | 35593 | 3302 | |
| 15 | 6.92 | C_16_H_18_O_9_ | 353.0878 | 353.0869 | -2.6 | 353.0884(16%) [M-H]^-^, 191.0573(70%), 179.0352(67%), 173.0455(100%), 161.0240(16%), 135.0468(51%), 93.0406(13%), 71.0175(6.3%) | 4-Caffeoylquinic acid | 77290 | 48981 | 10226 | |
| 16 | 6.94 | C_21_H_26_O_13_ | 485.1301 | 485.1281 | -4.1 | 485.1220(7.8%) [M-H]^-^, 177.0199(100%) | 7-Hydroxy coumarin-6-O-rutinoside | ND | 1212 | 2743 | |
| 17 | 6.94 | C_21_H_20_O_10_ | 431.0984 | 431.0969 | -3.5 | 431.0961(100%) [M-H]^-^, 311.0561(81%), 293.0473(13%), 283.0614(71%), 255.0656(10%), 227.0675(5.0%) | Isovitexin | 1754 | 3651 | 8107 | |
| 18 | 7.01 | C_21_H_26_O_12_ | 469.1352 | 469.1333 | -4.0 | 469.1349(8.6%) [M-H]^-^, 161.0248(100%), 133.0290(5.2%) | Coumarin-7-O-rutinoside | ND | 1221 | 7660 | |
| 19 | 7.44 | C_15_H_20_O_9_ | 343.1035 | 343.1027 | -2.2 | 343.1023(15%) [M-H]^-^, 299.1105(30%), 284.0913(5.0%), 212.1017(15%), 197.0446(100%), 182.0246(45%), 138.0341(50%), 123.0133(25%), 95.0268(4.9%), 71.0183(4.9%) | Dihydrocaffeic acid-glc | 9432 | ND | 4223 | |
| 20 | 7.56 | C_18_H_26_O_10_ | 401.1453 | 401.1440 | -3.3 | 401.1421(100%) [M-H]^-^, 269.1021(82%), 233.0659(28%), 161.0477(46%), 113.0295(18%), 101.0262(55%) | Icariside F2 | 21006 | ND | 8956 | |
| 21 | 7.69 | C_21_H_26_O_13_ | 485.1301 | 485.1281 | -4.1 | 485.1272(10.4%) [M-H]^-^, 177.0197(100%) | 6-Hydroxy coumarin-7-O-rutinoside | ND | 1817 | 24343 | |
| 22 | 8.08 | C_24_H_22_O_14_ | 533.0937 | 533.0916 | -4.0 | 533.0967(4.9%) [M-H]^-^, 489.2301(25%), 447.2366(9.8%), 429.2124(40%), 411.2043(4.9%), 371.0643(30%), 209.0304(100%), 191.0223(25%), 179.0602(15%), 113.0264(5.0%), 85.0353(4.9%) | Dicaffeoyl altraric acid | ND | 76935 | 2738 | |
| 23 | 8.20 | C_15_H_20_O_4_ | 265.1434 | 265.1427 | -2.8 | 265.1754(32%) [M+H]^+^, 187.1076(32%), 175.0798(65%), 91.0580(100%) | 4,15-Epoxy-8β-hydroxyasterolide | 57 | ND | 449 | |
| 24 | 8.22 | C_21_H_30_O_14_ | 505.1563 | 505.1542 | -4.1 | 505.1510(51%) [M-H]^-^, 373.0923(16%), 197.0457(100%), 182.0195(33%) | 3,4-Di-O-glc-benzenepropanoic acid | 1112 | ND | 907 | |
| 25 | 8.23 | C_27_H_30_O_16_ | 609.1461 | 609.1435 | -4.2 | 609.1422(100%) [M-H]^-^, 519.1212(13%), 489.1055(47%), 369.0611(27%), 341.0673(13%) | Kaempferol 3,7-diglucoside | ND | 7762 | 1871 | |
| 26 | 8.24 | C_16_H_18_O_8_ | 337.0929 | 337.0918 | -3.2 | 337.0960(8.2%) [M-H]^-^, 191.0553(100%), 129.0576(8.2%), 93.0385(17%) | 5-p-Coumaroylquinic acid | 7495 | 4588 | 2995 | |
| 27 | 8.48 | C_16_H_18_O_8_ | 337.0929 | 337.0915 | -4.0 | 337.0147(20%) [M-H]^-^, 173.0475(100%), 163.0450(20%) | 4-p-Coumaroylquinic acid | 896 | 24273 | 1064 | |
| 28 | 8.60 | C_9_H_6_O_4_ | 177.0193 | 177.0199 | 3.2 | 177.0211(100%) [M-H]^-^, 133.0318(44%), 121.0380(12%), 109.0322(12%), 91.0242(19%), 89.0429(31%), 77.0486(12%), 65.0129(12%) | 6,7-Dihydroxy coumarin | ND | 853 | 4192 | |
| 29 | 8.92 | C_24_H_22_O_14_ | 533.0937 | 533.0910 | -5.0 | 533.0857(20%) [M-H]^-^, 371.0582(39%), 209.0294(100%), 191.0220(20%) | Dicaffeoyl altraric acid | ND | 32326 | 653 | |
| 30^*^ | 9.08 | C_27_H_30_O_15_ | 593.1512 | 593.1492 | -3.3 | 593.1499(100%) [M-H]^-^, 503.1179(6.5%), 473.1055(21%), 383.0742(16%), 353.0640(24%) | Vicenin II | ND | 916289 | 32992 | |
| 31 | 9.19 | C_17_H_20_O_9_ | 367.1035 | 367.1022 | -3.5 | 367.1048(8.5%) [M-H]^-^, 193.0485(17%), 191.0554(100%), 173.0434(17%), 134.0356(8.5%), 93.0395(17%) | 5-O-Feruloylquinic acid | 10543 | 22750 | 4785 | |
| 32 | 9.32 | C_17_H_20_O_9_ | 367.1035 | 367.1021 | -4.3 | 367.1047(8.1%) [M-H]^-^, 193.0560(41%), 173.0480(100%), 137.0290(16%), 93.0373(16%), 59.0302(8.1%) | 4-Feruloylquinic acid | 3102 | 333851 | 4053 | |
| 33 | 9.61 | C_9_H_6_O_3_ | 161.0244 | 161.0254 | 5.9 | 161.0249(100%) [M-H]^-^, 133.0297(62%), 117.0326(12%), 105.0375(17%), 89.0401(12%), 77.0441(15%), 65.0109(7.6%) | 6-Hydroxycoumarin | 2503 | 7605 | 10947 | |
| 34 | 9.92 | C_28_H_32_O_16_ | 625.1763 | 625.1752 | -1.8 | 625.1767(100%) [M+H]^+^, 607.1594(62%), 553.1393(38%), 505.1284(50%), 487.1224(87%), 439.1009(74%), 355.0838(50%) | Diosmetin-6,8-di-C-glucoside | ND | 71232 | 7058 | |
| 35 | 9.97 | C_24_H_22_O_14_ | 533.0937 | 533.0912 | -4.7 | 533.0969(32%) [M-H]^-^, 371.0559(100%), 353.0479(32%), 209.0306(100%), 191.0201(100%) | Dicaffeoyl altraric acid | ND | 34307 | 616 | |
| 36 | 10.24 | C_10_H_8_O_4_ | 191.0350 | 191.0352 | 1.2 | 191.0364(8.9%) [M-H]^-^, 176.0123(100%), 148.0179(55%), 120.0256(23%), 104.0308(55%),92.9975(4.4%) | Scopoletin | 4249 | 1775 | 5124 | |
| 37 | 10.39 | C_24_H_22_O_14_ | 533.0937 | 533.0914 | -4.2 | 533.0870(24%) [M-H]^-^, 371.583(49%), 209.0311(100%), 191.0215(24%), 164.8935(24%), 133.0269(24%), 85.0355(24%) | Dicaffeoyl altraric acid | ND | 31781 | 675 | |
| 38 | 10.45 | C_10_H_10_O_4_ | 193.0506 | 193.0509 | 1.6 | 193.0474(28%) [M-H]^-^, 178.0233(44%), 158.8460(28%), 134.0403(100%) | Ferulic acid | ND | 39714 | 1607 | |
| 39 | 10.52 | C_21_H_20_O_10_ | 431.0984 | 431.0966 | -4.0 | 431.1000(100%) [M-H]^-^, 311.0589(61%), 283.0610(39%) | Vitexin isomer | ND | 834 | 1586 | |
| 40 | 10.87 | C_24_H_22_O_14_ | 533.0937 | 533.0913 | -4.4 | 533.0847(8.9%) [M-H]^-^, 371.0591(46%), 209.0297(100%), 191.0181(37%) | Dicaffeoyl altraric acid | ND | 82479 | 1867 | |
| 41 | 11.16 | C_33_H_42_O_19_ | 741.2248 | 741.2233 | -1.9 | 741.2249(100%) [M-H]^-^, 621.1658(13%), 271.0614(9.0%), 151.0049(4.2%) | Eriodictyol-7-O-glc-rha-rha | ND | 97892 | 37787 | |
| 42^*^ | 11.24 | C_27_H_32_O_15_ | 595.1668 | 595.1649 | -3.2 | 595.1665(30%) [M-H]^-^, 287.0560(100%), 151.0051(29%), 135.0463(6.8%) | Eriocitrin | ND | 210507 | 145294 | |
| 43 | 11.37 | C_21_H_20_O_10_ | 431.0984 | 431.0968 | -3.7 | 431.0962(76%) [M-H]^-^, 341.0687(24%), 311.0564(100%), 283.0644(24%) | Vitexin | ND | 6064 | 1017 | |
| 44 | 11.46 | C_21_H_22_O_11_ | 449.1089 | 449.1069 | -4.4 | 449.1087(12%) [M-H]^-^, 329.0661(7.8%), 287.0540(100%), 193.0143(3.9%), 175.0109(3.9%), 151.0042(52%), 135.0494(16%), 65.0059(3.9%) | Sinensin | ND | 3967 | 3499 | |
| 45 | 11.47 | C_24_H_22_O_14_ | 533.0937 | 533.0908 | -5.4 | 533.0908(16%) [M-H]^-^, 209.0308(100%) | Dicaffeoyl altraric acid | ND | 44959 | 766 | |
| 46 | 11.60 | C_21_H_20_O_10_ | 431.0984 | 431.0963 | -4.8 | 431.0886(33%) [M-H]^-^, 341.0689(33%), 323.0634(33%), 311.0550(100%), 283.0581(67%), 269.0398(33%), 223.0042(16%) | Vitexin isomer | ND | 4867 | 1124 | |
| 47^*^ | 11.78 | C_27_H_32_O_15_ | 595.1668 | 595.1651 | -2.9 | 595.1645(100%) [M-H]^-^, 459.1129(72%), 339.0721(47%), 287.0552(9.6%), 235.0251(8.2%), 151.0051(30%), 135.0469(10%) | Neoeriocitrin | ND | 1218108 | 692935 | |
| 48 | 11.92 | C_27_H_30_O_16_ | 609.1461 | 609.1443 | -3.0 | 609.1481(100%) [M-H]^-^, 301.0383(51%) | Quercetin-7-O-rutinoside | ND | ND | 2335 | |
| 49 | 11.94 | C_27_H_30_O_16_ | 611.1607 | 611.1591 | -2.5 | 611.1987(31%) [M+H]^+^, 465.1061(10%), 355.071(30%), 303.0491(100%), 266.9986(20%), 221.0880(9.8%) | Rutin | ND | 22387 | 1685 | |
| 50 | 12.10 | C_34_H_44_O_20_ | 771.2353 | 771.2341 | -1.6 | 771.2335(100%) [M-H]^-^, 301.0713(11%) | Hesperetin-7-O-glc-glc-rha | ND | 151461 | 61244 | |
| 51 | 12.26 | C_22_H_22_O_11_ | 463.1235 | 463.1225 | -2.2 | 463.1398(33%) [M+H]^+^, 343.0785(84%), 325.0653(16%), 313.0681(100%), 300.0513(51%) | Diosmetin-8-C-glucoside | ND | 18563 | 558 | |
| 52 | 12.40 | C_22_H_22_O_11_ | 463.1235 | 463.1224 | -2.4 | 463.1223(100%) [M+H]^+^, 445.1130(39%), 427.1010(35%), 343.0801(89%), 313.0703(82%) | Chrysoeriol-8-c-glucoside | ND | 20747 | 5032 | |
| 53 | 12.63 | C_33_H_42_O_18_ | 725.2298 | 725.2288 | -1.5 | 725.2293(100%) [M-H]^-^, 605.1720(13%), 271.0598(11%), 151.0030(4.2%) | Naringenin-7-O-glc-rha-rha | ND | 26954 | 20178 | |
| 54^*^ | 12.92 | C_27_H_32_O_14_ | 579.1719 | 579.1700 | -3.3 | 579.1689(13%) [M-H]^-^, 271.0603(100%), 151.0050(7.6%) | Narirutin | ND | 1957451 | 367175 | |
| 55 | 13.39 | C_27_H_30_O_14_ | 577.1563 | 577.1541 | -3.7 | 577.1547(22%) [M-H]^-^, 269.0456(100%) | Rhoifolin isomer | ND | 34087 | 3798 | |
| 56 | 13.44 | C_23_H_24_O_13_ | 507.1144 | 507.1123 | -4.2 | 507.1123(100%) [M-H]^-^, 492.0901(12%), 344.0553(54%), 329.0294(92%), 301.0316(27%), 286.0105(12%) | Syringetin-3-O-glucoside | ND | 36893 | 6795 | |
| 57^*^ | 13.60 | C_21_H_22_O_10_ | 433.1140 | 433.1124 | -3.8 | 271.0608(100%) [M-H]^-^, 151.0042(16%), 119.0534(5.1%) | Naringenin-7-O-glucoside | ND | 48845 | 14184 | |
| 58^*^ | 13.72 | C_27_H_32_O_14_ | 579.1719 | 579.1702 | -3.0 | 579.1688(100%) [M-H]^-^, 459.1135(51%), 313.0715(7.9%), 271.0604(76%), 235.0242(6.7%), 151.0047(29%) | Naringin | ND | 3645243 | 2037252 | |
| 59 | 13.86 | C_34_H_44_O_19_ | 755.2404 | 755.2393 | -1.4 | 755.2392(100%) [M-H]^-^, 301.0705(9.3%) | Hesperetin-7-O-glc-rha-rha | ND | 195082 | 74452 | |
| 60 | 13.89 | C_23_H_24_O_13_ | 507.1144 | 507.1128 | -3.1 | 507.1116(100%) [M-H]^-^, 492.0910(15%), 344.0482(33%), 329.0286(72%), 301.0310(19%), 258.0196(9.3%) | Syringetin-3-O-galactoside | ND | 10436 | 6452 | |
| 61 | 14.15 | C_27_H_30_O_14_ | 577.1563 | 577.1549 | -2.4 | 577.1555(100%) [M-H]^-^, 269.0454(79%) | Rhoifolin | ND | 51802 | 32376 | |
| 62^*^ | 14.43 | C_28_H_34_O_15_ | 609.1825 | 609.1806 | -3.1 | 609.1791(13%) [M-H]^-^, 301.0706(100%) | Hesperidin | ND | 2290056 | 619366 | |
| 63 | 14.57 | C_28_H_32_O_15_ | 607.1668 | 607.1652 | -2.7 | 607.1670(38%) [M-H]^-^, 299.0561(100%), 297.0395(5.2%), 284.0305(20%) | Diosmetin 7-O-Neohesperidoside | ND | 29751 | 10832 | |
| 64 | 14.94 | C_28_H_32_O_15_ | 607.1668 | 607.1651 | -2.9 | 607.1649(100%) [M-H]^-^, 299.0554(47%), 284.0333(10%) | Diosmetin-7-O-rutinoside isomer | ND | 115843 | 5444 | |
| 65^*^ | 15.38 | C_28_H_34_O_15_ | 609.1825 | 609.1807 | -2.9 | 609.1796(100%) [M-H]^-^, 489.1382(6.9%), 343.0811(7.1%), 301.0701(58%), 286.0476(5.4%) | Neohesperidin | ND | 3542458 | 2441424 | |
| 66^*^ | 15.43 | C_28_H_32_O_15_ | 607.1668 | 607.1659 | -1.6 | 607.1654(73%) [M-H]^-^, 299.0551(100%), 284.0311(22%) | Diosmin | ND | 58504 | 33054 | |
| 67 | 15.50 | C_22_H_24_O_d_ | 463.1246 | 463.1232 | -2.9 | 301.0711(100%) [M-H]^-^, 286.0463(7.8%), 242.0576(5.6%), 164.0126(4.5%) | Hesperetin-5-O-glucoside | ND | 21723 | 8689 | |
| 68 | 16.36 | C_22_H_24_O_11_ | 463.1246 | 463.1223 | -5.0 | 463.1213(38%) [M-H]^-^, 301.0705(100%), 286.0503(9.4%), 257.0790(9.4%), 240.0379(9.3%), 199.0391(14%), 164.0065(9.3%), 151.0124(4.7%) | Hesperetin-7-O-glucoside | ND | 1355 | 3115 | |
| 69 | 18.15 | C_15_H_12_O_6_ | 287.0561 | 287.0559 | -0.7 | 287.0500(14%) [M-H]^-^, 266.9734(4.7%), 182.9884(4.7%), 165.0224(4.7%), 151.0056(38%), 135.0460(100%), 107.0238(9.3%), 83.0200(9.3%), 65.0094(9.3%) | Eriodictyol | ND | 9871 | 1841 | |
| 70 | 18.58 | C_16_H_12_O_5_ | 283.0612 | 283.0607 | -1.8 | 283.0600(30%) [M-H]^-^, 268.0358(100%), 239.0342(25%), 211.0399(25%), 195.0461(20%), 184.0538(10%), 135.0090(10%) | Acacetin | 556 | 651 | 1715 | |
| 71^*^ | 21.10 | C_28_H_34_O_14_ | 593.1876 | 593.1858 | -3.0 | 593.1850(32%) [M-H]^-^, 309.0764(8.2%), 285.0767(100%) | Poncirin | ND | 840513 | 34525 | |
| 72 | 21.10 | C_16_H_14_O_5_ | 285.0769 | 285.0763 | -1.9 | 285.0752(100%) [M-H]^-^, 270.0491(20%), 243.0687(9.8%), 216.9878(9.8%), 200.0506(9.8%), 171.0504(9.8%), 164.0097(50%), 151.0043(30%), 136.0186(20%) | 4'-Methylnaringenin | ND | 24935 | 925 | |
| 73 | 22.48 | C_28_H_34_O_14_ | 593.1876 | 593.1860 | -2.6 | 593.1859(100%) [M-H]^-^, 473.1454(6.9%), 327.0840(6.8%), 285.0764(92%) | Hesperetin-7-rha-rha | ND | 12356 | 37514 | |
| 74 | 22.56 | C_18_H_16_O_6_ | 329.1020 | 329.1011 | -2.5 | 329.0980(73%) [M+H]^+^, 314.0852(28%), 299.0557(100%), 271.0620(37%), 195.0305(18%), 181.0127(18%), 174.9903(8.9%), 153.0141(18%), 121.0280(8.9%), 98.9882(45%), 80.9894(8.9%) | 3'-Hydroxy-5,7,4'-trimethoxyflavone | ND | 15731 | 1022 | |
| 75 | 23.44 | C_18_H_16_O_6_ | 329.1020 | 329.1015 | -1.5 | 329.1021(100%) [M+H]^+^, 314.0792(100%), 299.0559(50%), 271.0590(26%), 268.0741(45%), 266.0531(4.7%), 243.0600(7.1%), 209.0671(4.7%), 182.0159(12%), 164.0125(9.4%), 133.0645(14%), 98.9869(24%), 69.0087(2.3%) | 8-Hydroxy-5,7-dimethoxy-2-(4-methoxyphenyl)-4H-1-benzopyran-4-one | ND | 18851 | 2943 | |
| 76^*^ | 23.51 | C_15_H_12_O_5_ | 271.0612 | 271.0609 | -1.0 | 271.0607(91%) [M-H]^-^, 227.0731(5.1%), 187.0450(7.9%), 177.0195(18%), 151.0051(98%), 145.0322(21%), 119.0529(100%), 107.0158(34%), 93.0379(13%), 83.0185(11%), 65.0104(18%) | Naringenin | ND | 87318 | 13404 | |
| 77 | 24.15 | C_15_H_10_O_5_ | 269.0456 | 269.0449 | -2.5 | 269.0447(100%) [M-H]^-^, 248.9968(12%), 204.9862(12%), 183.0416(12%), 154.9969(24%), 133.0324(12%), 116.9953(38%) | Apigenin | ND | 216 | 421 | |
| 78 | 24.66 | C_36_H_53_N_7_O_9_ | 728.3978 | 728.3968 | -1.4 | 728.3961(100%) [M+H]^+^, 700.3989(33%), 615.3197(11%), 587.3211(16%), 502.2258(11%), 282.1406(7.9%) | Citrusin III | ND | 31440 | 4485 | |
| 79^*^ | 25.50 | C_16_H_14_O_6_ | 301.0718 | 301.0716 | -0.6 | 301.0715(100%) [M-H]^-^, 286.0486(33%), 257.0807(11%), 242.0574(25%), 201.0187(14%), 188.0490(4.5%), 174.0332(12%), 164.0123(60%), 151.0045(27%), 136.0174(22%), 108.0240(14%), 80.0314(4.4%) | Hesperetin | ND | 22777 | 22250 | |
| 80 | 25.69 | C_20_H_36_O_7_ | 389.2534 | 389.2539 | 1.3 | 389.2536(100%) [M+H]^+^, 330.2064(40%), 327.2066(23%), 232.1690(50%) | Tetradecyl citric acid | 6385 | ND | 6176 | |
| 81 | 25.74 | C_16_H_12_O_6_ | 299.0561 | 299.0558 | -1.2 | 299.0501(40%) [M-H]^-^, 284.0344(100%), 278.9918(20%), 190.9936(20%), 141.0000(9.8%) | 7-O-Methylluteolin | ND | 1577 | 670 | |
| 82 | 27.31 | C_19_H_18_O_7_ | 359.1125 | 359.1117 | -2.2 | 359.1113(51%) [M+H]^+^, 344.0889(29%), 329.0652(100%), 286.0450(15%), 257.0453(9.0%), 183.0297(7.3%), 168.0027(3.6% | 5,7-Dihydroxy-8,3',4'-trimethoxyflavone | ND | 55334 | 3820 | |
| 83 | 27.50 | C_20_H_20_O_7_ | 373.1282 | 373.1276 | -1.6 | 373.1279(72%) [M+H]^+^, 358.1050(14%), 343.0812(100%), 310.0866(11%), 181.0132(6.2%) | 3',4',5,7,8-Pentamethoxyflavone | ND | 9236 | 75190 | |
| 84 | 27.61 | C_34_H_53_N_7_O_9_ | 704.3978 | 704.3963 | -2.1 | 704.3988(100%) [M+H]^+^, 686.3872(90%), 573.2999(26%), 555.2923(14%), 502.2616(17%), 306.1474(14%) | Citrusin Ⅰ | ND | 1468 | 9363 | |
| 85 | 27.89 | C_20_H_20_O_8_ | 389.1231 | 389.1223 | -2.0 | 389.1234(64%) [M+H]^+^, 374.1013(26%), 359.0761(100%), 313.0719(18%), 211.0232(5.3%), 183.0297(4.3%) | 5-Hydroxy-3,7,8,3',4'-pentamethoxyflavone | ND | 708 | 8316 | |
| 86 | 28.28 | C_15_H_20_O_3_ | 249.1485 | 249.1481 | -1.9 | 249.1447(50%) [M+H]^+^, 232.1444(100%), 217.1217(29%), 204.1500(43%), 189.1267(58%), 147.0788(86%), 133.0675(79%), 105.0710(50%), 91.0559(57%), 79.0578(43%) | 13-hydroxyl-atractylenolide II | 23307 | ND | 12446 | |
| 87 | 28.42 | C_21_H_22_O_9_ | 419.1337 | 419.1328 | -2.1 | 419.1337(97%) [M+H]^+^, 404.1097(21%), 389.0869(100%), 371.0750(13%), 358.1087(5.8%), 313.0404(2.8%), 241.0742(2.8%), 211.0263(20%), 183.0293(11%), 165.0177(4.3%) | 8-Hydroxy-3,5,7,3',4',5'-hexamethoxyflavone | ND | 1367 | 10308 | |
| 88 | 28.43 | C_21_H_22_O_8_ | 403.1387 | 403.1379 | -2.0 | 403.1391(100%) [M+H]^+^, 388.1158(13%), 373.0920(58%), 345.0959(7.2%), 327.0853(26%), 165.0537(4.1%) | 3,5,7, 3',4',5'-Hexamethoxyflavone | ND | 450359 | 10606 | |
| 89^*^ | 28.53 | C_18_H_16_O_8_ | 359.0772 | 359.0759 | -3.8 | 359.0756(26%) [M-H]^-^, 344.0521(89%), 329.0296(100%), 301.0345(35%) | 5,7,3'-Trihydroxy-6,4',5'-trimethoxyflavone | ND | 14465 | ND | |
| 90 | 28.91 | C_20_H_20_O_7_ | 373.1282 | 373.1276 | -1.6 | 373.1283(100%) [M+H]^+^, 357.0967(24%), 343.0809(60%), 329.1021(24%), 312.0995(37%), 153.0176(4.5%) | 3,3',4',5,7-Pentamethylquercetin | ND | 8275 | 2854 | |
| 91 | 28.98 | C_17_H_14_O_6_ | 315.0863 | 315.0856 | -1.8 | 315.0864(100%) [M+H]^+^, 299.0550(100%), 271.0592(86% | 3,4'-Di-O-methylkaempferol | ND | 21881 | 7446 | |
| 92 | 29.15 | C_20_H_20_O_8_ | 389.1231 | 389.1225 | -1.6 | 389.1225(72%) [M+H]^+^, 374.1006(13%), 359.0755(100%), 331.0815(6.4%), 257.0761(2.1%), 165.0532(6.9%), 149.0217(2.1%) | 3,7,3',4',5'-Penta-O-methylmyricetin | ND | 1548 | 15506 | |
| 93^*^ | 29.23 | C_26_H_30_O_8_ | 469.1868 | 469.1856 | -2.5 | 469.1854(100%) [M-H]^-^, 321.1104(12%), 278.1326(12%), 229.1258(14%) | Limonin | ND | 161958 | 9004 | |
| 94 | 29.86 | C_21_H_22_O_8_ | 403.1387 | 403.1379 | -2.0 | 403.1379(100%) [M+H]^+^, 387.1089(21%), 373.0910(50%), 370.1109(14%), 284.0662(6.9%), 165.0696(2.2%) | 5,7,8,3',4',5'-Hexamethoxyflavone | ND | 232786 | 4829 | |
| 95^*^ | 29.94 | C_18_H_16_O_5_ | 313.1071 | 313.1063 | -2.6 | 313.1062(100%) [M+H]^+^, 298.0844(62%), 268.0789(36%), 255.0651(17%), 227.0706(8.5%), 133.0681(6.5%) | 4',5,7-Trimethoxyflavone | ND | 1030 | 2771 | |
| 96 | 30.38 | C_21_H_22_O_8_ | 403.1387 | 403.1383 | -1.1 | 403.1383(31%) [M+H]^+^, 388.1149(18%), 373.0912(100%), 327.0861(9.4%), 211.0236(4.2%), 183.0285(3.7%) | 3,5,7,8,3',4'-Hexamethoxyflavone | ND | 4539439 | 305079 | |
| 97 | 30.42 | C_19_H_18_O_6_ | 343.1176 | 343.1175 | -0.3 | 343.1173(100%) [M+H]^+^, 327.0849(23%), 313.0699(72%), 299.0913(36%), 285.0752(20%), 282.0882(59%), 153.0190(8.9%) | 3',4'-Dimethoxyluteolin | ND | 8107 | 3044 | |
| 98 | 30.60 | C_19_H_18_O_8_ | 375.1074 | 375.1066 | -2.4 | 375.1074(100%) [M+H]^+^, 360.0892(17%), 345.0576(72%), 327.0469(22%), 302.0411(11%), 227.04965.4%), 197.0075(17%), 169.0157(5.5%), 113.0210(5.5%) | 5,6-Dihydroxy-3',4',7,8-tetramethoxyflavone | ND | 379 | 1848 | |
| 99 | 30.72 | C_20_H_20_O_8_ | 389.1231 | 389.1224 | -1.8 | 389.1229(100%) [M+H]^+^, 356.0860(47%), 331.0798(39%), 316.0556(8.1%), 313.0706(26%), 297.0768(4.9%), 165.0561(4.9%) | 7-Hydroxy-3,5,3',4',5'-pentamethoxyflavone | ND | 844 | 8094 | |
| 100 | 31.03 | C_15_H_18_O_2_ | 231.1380 | 231.1372 | -3.3 | 231.1367(58%) [M+H]^+^, 213.1251(24%), 163.0744(82%), 91.0564(100%) | Atractylenolide Ⅰ isomer | 13499 | ND | 8655 | |
| 101^*^ | 31.03 | C_15_H_20_O_3_ | 249.1485 | 249.1478 | -3.0 | 249.1461(2.5%) [M+H]^+^, 231.1365(87%), 213.1277(18%), 175.0752(54%), 163.0764(100%), 161.0602(46%), 105.0710(41%), 91.0564(41%) | Atractylenolide III | 15735 | ND | 10161 | |
| 102^*^ | 31.27 | C_22_H_24_O_9_ | 433.1493 | 433.1488 | -1.3 | 433.1493(53%) [M+H]^+^, 418.1264(19%), 403.1022(100%), 385.0921(12%), 373.0555(4.3%), 165.0538(2.2%) | 3,5,6,7,8,3',4'-Heptamethoxyflavone | ND | 4831 | 196516 | |
| 103 | 31.71 | C_19_H_18_O_7_ | 359.1125 | 359.1121 | -1.2 | 359.1128(100%) [M+H]^+^, 343.0812(39%), 314.0804(11%), 298.0826(83%), 165.0559(5.5%), 162.0684(22%) | 5,8-Dihydroxy-3',4',7-trimethoxyflavone | ND | 807 | 2754 | |
| 104^*^ | 31.77 | C_20_H_20_O_7_ | 373.1282 | 373.1279 | -0.9 | 373.1281(29%) [M+H]^+^, 358.1047(18%), 343.0810(100%), 297.0757(12%), 211.0234(6.0%) | Tangeretin | ND | 101766 | 174426 | |
| 105 | 31.84 | C_21_H_22_O_9_ | 419.1337 | 419.1331 | -1.3 | 419.1334(51%) [M+H]^+^, 404.1100(24%), 389.0861(100%), 371.0757(10%), 361.0915(24%), 331.0447(4.3%), 165.0540(2.6%) | 4'-Hydroxy-3,5,6,7,8,3'-hexamethoxyflavone | ND | 5201716 | 170980 | |
| 106 | 31.95 | C_15_H_19_NO | 230.1539 | 230.1533 | -2.9 | 230.1539(100%) [M+H]^+^, 215.1289(7.8%), 174.0910(12%), 160.0757(12%) | Atractylenolactam | 5532 | ND | 4649 | |
| 107 | 32.29 | C_15_H_20_O_4_ | 265.1434 | 265.1429 | -1.9 | 265.1221(8.9%) [M+H]^+^, 231.1368(54%), 205.1214(18%), 185.1343(37%), 163.0756(100%), 161.0597(37%), 159.1178(37%), 135.041137%), 119.0885(37%), 91.0553(18%) | Atractylenolide Ⅴ | 1384 | ND | 977 | |
| 108 | 32.37 | C_19_H_18_O_8_ | 375.1074 | 375.1062 | -3.3 | 375.1084(100%) [M+H]^+^, 360.0781(60%), 327.0487(30%), 315.0426(20%), 269.9940(10%), 186.9956(9.8%), 151.0769(9.8%) | 5,7-Dihydroxy-6,8,3',4'-tetramethoxyflavone | ND | 129 | 1108 | |
| 109 | 32.73 | C_20_H_20_O_9_ | 405.1180 | 405.1174 | -1.6 | 405.1173(100%) [M+H]^+^, 375.0705(61%), 357.0595(27%), 347.0778(51%), 332.0500(15%), 329.0687(7.4%), 270.0551(4.8%), 227.0561(4.8%), 163.0352(2.4%) | 3,3'-Dihydroxy-5,6,7,8,4'-pentamethoxyflavone | ND | 951 | 4142 | |
| 110 | 32.76 | C_21_H_22_O_8_ | 403.1387 | 403.1380 | -1.8 | 403.1380(77%) [M+H]^+^, 373.0896(100%), 355.0776(13%), 315.0496(13%), 299.0913(5.8%), 256.0702(3.7%), 211.0227(7.6%), 183.0264(3.7%), 135.0458(5.8%) | 5,6,7,8,3',4'-Hexamethoxyflavone | ND | 270683 | 29804 | |
| 111^*^ | 32.90 | C_20_H_20_O_8_ | 389.1231 | 389.1225 | -1.6 | 389.1236(100%) [M+H]^+^, 374.0987(18%), 359.0755(81%), 356.0891(10%), 341.0653(36%), 331.0815(6.3%), 169.0099(10%) | 5-Hydroxy-3',4',6,7,8-tetramethoxyflavone | ND | 8166 | 30141 | |
| 112 | 33.31 | C_20_H_20_O_8_ | 389.1231 | 389.1224 | -1.7 | 389.1222(66%) [M+H]^+^, 374.0992(23%), 359.0767(100%), 331.0799(28%), 298.0468(16%), 270.0558(5.5%), 135.0439(4.5%) | 6-Hydroxy-3',4',5,7,8-tetramethoxyflavone | ND | 2902 | 25643 | |
| 113 | 33.67 | C_15_H_20_O_2_ | 233.1536 | 233.1529 | -2.9 | 233.1535(66%)[M+H]^+^, 215.1436(56%), 187.1476(98%), 159.0802(69%), 131.0855(100%), 105.0714(72%), 91.0562(46%) | Atractylenolide II isomer | 22827 | ND | 19100 | |
| 114^*^ | 33.86 | C_15_H_20_O_2_ | 233.1536 | 233.1529 | -3.1 | 233.1530(55%) [M+H]^+^, 215.1421(55%), 187.474(87%), 177.0903(54%), 159.0801(62%), 151.0753(63%), 145.1011(64%), 131.0860(100%), 105.0713(85%), 91.0564(64%) | Atractylenolide II | 76018 | ND | 56825 | |
| 115 | 34.17 | C_15_H_20_O_3_ | 249.1485 | 249.1478 | -2.8 | 249.1487(64%) [M+H]^+^, 232.1453(100%), 189.1280(63%), 133.0659(100%), 93.0712(82%) | 4(R),15-Epoxy-atractylenolide II | 28754 | ND | 17991 | |
| 116 | 34.32 | C_17_H_24_O_3_ | 277.1798 | 277.1790 | -2.9 | 277.2232(8.1%) [M+H]^+^, 203.1068(13%), 165.0539(33%), 147.0452(100%), 91.0554(17%), 57.0753(25%) | 8β-Ethoxyasterolid | 3450 | ND | 4348 | |
| 117 | 35.14 | C_16_H_22_O_3_ | 263.1642 | 263.1635 | -2.7 | 263.1529(5.7%) [M+H]^+^, 245.1572(5.6%), 231.1371(94%), 189.0921(43%), 163.0755(100%), 105.0711(32%) | 8β-Methoxyatractylenolide | 27858 | ND | 9271 | |
| 118^*^ | 35.44 | C_15_H_18_O_2_ | 231.1380 | 231.1374 | -2.5 | 231.1383(100%) [M+H]^+^, 213.1271(10%), 185.1327(50%), 157.1015(46%), 143.0863(45%) | Atractylenolide Ⅰ | 77661 | ND | 53540 | |
| 119 | 36.21 | C_15_H_22_O | 219.1743 | 219.1738 | -2.5 | 219.1738(52%) [M+H]^+^, 201.1632(26%), 145.1013(69%), 95.0878(100%), 91.0566(71%) | Selina-4(14),7(11)-dien-8-one | 92850 | ND | 46331 | |
| 120 | 36.39 | C_15_H_20_O_2_ | 233.1536 | 233.1528 | -3.4 | 233.1526(83%) [M+H]^+^, 215.1432(40%), 187.1480(69%), 159.1171(69%), 145.1018(78%), 131.0857(100%), 105.0715(70%), 95.0885(53%), 91.0571(39%) | 3β-Hydroxyatractylone | 22827 | ND | 11704 | |
| 121 | 36.46 | C_17_H_22_O_4_ | 291.1591 | 291.1585 | -2.0 | 291.1577(39%) [M+H]^+^, 231.1380(62%), 185.1311(100%), 157.1019(85%), 142.0796(73%), 128.0620(74%), 91.0582(27%) | 3β-Acetoxyl atractylenolide | 1026 | ND | 2286 | |
| 122 | 36.46 | C_17_H_22_O_3_ | 275.1642 | 275.1637 | -1.6 | 275.1645(100%) [M+H]^+^, 215.1433(62%), 157.1013(31%), 145.1014(63%) | 3β-Acetoxyatractylone | 19908 | ND | 14381 | |
| 123 | 39.00 | C_15_H_22_ | 203.1794 | 203.1790 | -2.0 | 203.1787(37%) [M+H]^+^, 147.1167(52%), 133.1016(49%), 105.0716(100%), 95.0877(54%), 91.0567(53%) | Atractylenolide Ⅵ | 14747 | ND | 7432 | |
| 124 | 39.11 | C_15_H_20_O_2_ | 233.1536 | 233.1529 | -3.0 | 233.1533(87%) [M+H]^+^, 215.1422(33%), 187.1480(66%), 159.1168(100%), 145.1020(75%), 131.0861(91%), 105.0718(46%), 95.0876(52%), 91.0571(50%) | Isoasterolide A | 12263 | ND | 20358 | |
| 125^*^ | 39.12 | C_15_H_20_O | 217.1587 | 217.1581 | -2.9 | 217.1584(100%) [M+H]^+^, 199.1481(19%), 143.0862(26%), 95.0515(38%) | Atractylone | 5664 | ND | 2535 | |
| 126 | 39.74 | C_18_H_32_O_2_ | 279.2330 | 279.2334 | 1.5 | 279.2324(100%) [M-H]^-^, 261.2216(1.7%) | Linoleic acid | 82167 | 98130 | 59076 | |
| 127 | 40.64 | C_16_H_32_O_2_ | 255.2330 | 255.2330 | 1.7 | 255.2331(100%) [M-H]^-^, 237.2144(2.4%) | Palmitic acid | 26609 | 78631 | 24997 | |
| 128 | 41.87 | C_15_H_22_ | 203.1794 | 203.1786 | -4.0 | 203.1792(40%) [M+H]^+^, 147.1167(58%), 133.1016(62%), 105.0711(100%) | Atractylenolide Ⅵ isomer | 3079 | ND | 26190 | |

(* indicates that the compound had been compared with the standard)

Table S4 Calibration curves, repeatability, precision, stability, recovery, LOQ and LOD results of 8 compounds in the samples by HPLC.

| Analyte | Calibration curves | Linear range  (μg•mL^-1^) | R^2^ | Repeatability RSD  (%) (n=6) | Precision RSD (%) (n=6) | | Stability  (n=6) | Recovery (%) (n=6) | | LOD | LOQ |
| --- | --- | --- | --- | --- | --- | --- | --- | --- | --- | --- | --- |
|  |  |  |  |  | Intra day | Inter day | RSD (%) | Average recovery | RSD | (μg·mL^-1^) | (μg·mL^-1^) |
| synephrine | y=3720.28*x-26041.48 | 26.05 - 1042.00 | 0.9998 | 2.7 | 0.7 | 2.8 | 2.1 | 99.20 | 1.74 | 0.58 | 1.76 |
| naringin | y=15878.69*x-12280.52 | 2.73 - 109.20 | 0.9961 | 1.1 | 2.6 | 1.5 | 2.2 | 102.07 | 1.11 | 0.62 | 2.08 |
| hesperidin | y=25281.72*x-26674.50 | 3.30 - 131.80 | 0.9960 | 2.7 | 1.1 | 2.0 | 1.9 | 97.61 | 2.55 | 0.22 | 0.74 |
| neohesperidin | y=20079.37*x-18023.59 | 2.84 - 113.40 | 0.9959 | 2.3 | 1.4 | 3.1 | 2.8 | 100.36 | 2.53 | 0.34 | 0.91 |
| atractylenolide Ⅰ | y=53100.50x-39212.49 | 2.63 - 105.20 | 0.9965 | 2.4 | 0.8 | 3.3 | 2.5 | 98.82 | 1.25 | 0.27 | 0.81 |
| atractylenolide Ⅱ | y=42557.45*x-17239.86 | 3.23 - 129.00 | 0.9969 | 1.2 | 2.4 | 2.7 | 1.6 | 101.94 | 2.49 | 0.13 | 0.38 |
| atractylenolide Ⅲ | y=29155.33*x-20521.11 | 2.69 - 107.40 | 0.9961 | 1.7 | 1.9 | 1.6 | 1.2 | 102.79 | 3.30 | 0.26 | 0.81 |
| atractylone | y=12811.93*x-33454.13 | 2.74 - 109.60 | 0.9976 | 2.8 | 2.2 | 3.8 | 1.3 | 99.56 | 1.24 | 0.78 | 2.28 |

Table S5 The 32 active compounds of PAMR.

| No. | Formula | Name | OB | DL | CAS | Structure |
| --- | --- | --- | --- | --- | --- | --- |
| 1 | C_15_H_16_O_9_ | Aesculin | 20.43% | 0.36 | 531-75-9 |  |
| 2 | C_16_H_18_O_9_ | 4-Caffeoylquinic acid | 24.50% | 0.33 | 905-99-7 |  |
| 3 | C_16_H_18_O_8_ | 3-O-p-Coumaroylquinic acid | 37.63% | 0.29 | 1899-30-5 |  |
| 4 | C_27_H_32_O_14_ | Naringin | 6.92% | 0.78 | 10236-47-2 |  |
| 5 | C_28_H_32_O_15_ | Diosmin | 12.70% | 0.66 | 520-27-4 |  |
| 6 | C_28_H_34_O_15_ | Hesperidin | 13.33% | 0.67 | 520-26-3 |  |
| 7 | C_21_H_20_O_10_ | Isovitexin | 31.29% | 0.72 | 38953-85-4 |  |
| 8 | C_21_H_20_O_10_ | Vitexin | 3.05% | 0.71 | 3681-93-4 |  |
| 9 | C_21_H_22_O_10_ | Naringenin-7-O-glucoside | 9.33% | 0.74 | 529-55-5 |  |
| 10 | C_15_H_10_O_5_ | Apigenin | 23.06% | 0.21 | 520-36-5 |  |
| 11 | C_15_H_12_O_5_ | Naringenin | 59.29% | 0.21 | 480-41-1 |  |
| 12 | C_15_H_12_O_6_ | Eriodictyol | 71.79% | 0.24 | 552-58-9 |  |
| 13 | C_16_H_12_O_5_ | Acacetin | 34.97% | 0.24 | 480-44-4 |  |
| 14 | C_16_H_12_O_6_ | 7-O-Methylluteolin | 36.47% | 0.27 | 20243-59-8 |  |
| 15 | C_16_H_14_O_6_ | Hesperetin | 70.31% | 0.27 | 520-33-2 |  |
| 16 | C_26_H_30_O_8_ | Limonin | 21.30% | 0.57 | 1180-71-8 |  |
| 17 | C_16_H_32_O_2_ | Palmitic acid | 19.30% | 0.10 | 57-10-3 |  |
| 18 | C_18_H_32_O_2_ | Linoleic acid | 41.90% | 0.14 | 60-33-3 |  |
| 19 | C_27_H_30_O_16_ | Rutin | 3.20% | 0.68 | 153-18-4 |  |
| 20 | C_18_H_16_O_5_ | 4',5,7-Trimethoxyflavone | 39.83% | 0.30 | 5631-70-9 |  |
| 21 | C_17_H_14_O_6_ | 3,4'-Di-O-methylkaempferol | 58.95% | 0.30 | 20869-95-8 |  |
| 22 | C_20_H_20_O_7_ | Tangeretin | 21.38% | 0.43 | 481-53-8 |  |
| 23 | C_21_H_22_O_8_ | 3,5,7,8,3',4'-Hexamethoxyflavone | 42.84% | 0.51 | 7741-47-1 |  |
| 24 | C_22_H_24_O_9_ | 3,5,6,7,8,3',4'-Heptamethoxyflavone | 23.91% | 0.58 | 1178-24-1 |  |
| 25 | C_15_H_18_O_2_ | Atractylenolide I | 37.37% | 0.15 | 73069-13-3 |  |
| 26 | C_15_H_20_O_3_ | Atractylenolide III | 68.11% | 0.17 | 73030-71-4 |  |
| 27 | C_15_H_20_O | Atractylone | 41.10% | 0.13 | 6989-21-5 |  |
| 28 | C_17_H_22_O_3_ | 3β-Acetoxyatractylone | 54.07% | 0.22 | 61206-10-8 |  |
| 29 | C_15_H_22_O | Selina-4(14),7(11)-dien-8-one | 32.31% | 0.10 | 54707-47-0 |  |
| 30 | C_17_H_24_O_3_ | 8β-Ethoxyasterolid | 35.95% | 0.21 | 113269-35-5 |  |
| 31 | C_9_H_13_NO_2_ | Synephrine | 79.00% | 0.04 | 94-07-5 |  |
| 32 | C_15_H_20_O_2_ | Atractylenolide II | 47.50% | 0.15 | 73069-14-4 |  |

Table S6 Predictive targets of the 32 active compounds

| No. | Name | Target | Symbol |
| --- | --- | --- | --- |
| 1 | Aesculin | Prostaglandin G/H synthase 2 | PTGS2 |
| 2 | 4-caffeoylquinic acid | Prostaglandin G/H synthase 2 | PTGS2 |
| 3 | 3-O-p-coumaroylquinic acid | Prostaglandin G/H synthase 1 | PTGS1 |
|  |  | Prostaglandin G/H synthase 2 | PTGS2 |
|  |  | Nuclear receptor coactivator 2 | NCOA2 |
| 4 | naringin | Cyclin-dependent kinase inhibitor 1 | CDKN1A |
|  |  | Tumor necrosis factor | TNFSF15 |
|  |  | Ras-specific guanine nucleotide-releasing factor 2 | RASGRF2 |
|  |  | RAF proto-oncogene serine/threonine-protein kinase | RAF1 |
| 5 | Diosmin | Prostaglandin G/H synthase 1 | PTGS1 |
|  |  | Prostaglandin G/H synthase 2 | PTGS2 |
|  |  | Trypsin-1 | PRSS1 |
|  |  | Nuclear receptor coactivator 2 | NCOA2 |
|  |  | Nuclear receptor coactivator 1 | NCOA1 |
| 6 | Hesperidin | Apoptosis regulator BAX | BAX |
|  |  | Caspase-3 | CASP3 |
|  |  | Prostaglandin G/H synthase 2 | PTGS2 |
|  |  | Intercellular adhesion molecule 1 | ICAM1 |
|  |  | Vascular cell adhesion protein 1 | VCAM1 |
|  |  | T-lymphoma invasion and metastasis-inducing protein 2 | TIAM2 |
| 7 | Isovitexin | Serotonin 3a | HTR3A |
|  |  | Prostaglandin G/H synthase 2 | PTGS2 |
|  |  | Androgen receptor | AR |
|  |  | Transcription factor p65 | RELA |
|  |  | Inhibitor of nuclear factor kappa-B kinase subunit beta | IKBKB |
|  |  | Tumor necrosis factor | TNFSF15 |
| 8 | Vitexin | Androgen receptor | AR |
|  |  | Prostaglandin G/H synthase 2 | PTGS2 |
|  |  | Mitogen-activated protein kinase 7 | MAPK7 |
|  |  | Choline/ethanolamine kinase | CHKB |
| 9 | Naringenin 7-O-glucoside | Prostaglandin G/H synthase 2 | PTGS2 |
| 10 | Apigenin | Prostaglandin G/H synthase 1 | PTGS1 |
|  |  | Androgen receptor | AR |
|  |  | Prostaglandin G/H synthase 2 | PTGS2 |
|  |  | Trypsin-1 | PRSS1 |
|  |  | Nuclear receptor coactivator 2 | NCOA2 |
|  |  | Sodium channel protein type 5 subunit alpha | SCN5A |
|  |  | Coagulation factor VII | F7 |
|  |  | Transcription factor p65 | RELA |
|  |  | RAC-alpha serine/threonine-protein kinase | AKT1 |
|  |  | Vascular endothelial growth factor A | VEGFA |
|  |  | G1/S-specific cyclin-D1 | CCND1 |
|  |  | Apoptosis regulator Bcl-2 | BCL2 |
|  |  | Bcl-2-like protein 1 | BCL2L1 |
|  |  | Proto-oncogene c-Fos | FOS |
|  |  | Cyclin-dependent kinase inhibitor 1 | CDKN1A |
|  |  | Eukaryotic translation initiation factor 6 | EIF6 |
|  |  | Apoptosis regulator BAX | BAX |
|  |  | Caspase-9 | CASP9 |
|  |  | Urokinase-type plasminogen activator | PLAU |
|  |  | Matrix metalloproteinase-9 | MMP9 |
|  |  | Retinoblastoma-associated protein | RB1 |
|  |  | Tumor necrosis factor | TNFSF15 |
|  |  | Transcription factor AP-1 | JUN |
|  |  | Eukaryotic translation elongation factor 1 epsilon-1 | EEF1E1 |
|  |  | Activator of 90 kDa heat shock protein ATPase homolog 1 | AHSA1 |
|  |  | Caspase-3 | CASP3 |
|  |  | Cellular tumor antigen p53 | TP63 |
|  |  | NF-kappa-B inhibitor alpha | NFKBIA |
|  |  | Ornithine decarboxylase | ODC1 |
|  |  | E3 ubiquitin-protein ligase Mdm2 | MDM2 |
|  |  | Interstitial collagenase | MMP1 |
|  |  | Hypoxia-inducible factor 1-alpha | HIF1A |
|  |  | Insulin-like growth factor 1 receptor | IGF1R |
|  |  | Protein CBFA2T1 | RUNX1T1 |
|  |  | Acetyl-CoA carboxylase 1 | ACACA |
|  |  | Heme oxygenase 1 | HMOX1 |
|  |  | Intercellular adhesion molecule 1 | ICAM1 |
|  |  | Induced myeloid leukemia cell differentiation protein Mcl-1 | MCL1 |
|  |  | G1/S-specific cyclin-D2 | CCND2 |
|  |  | Interleukin-2 | IL2 |
|  |  | G2/mitotic-specific cyclin-B1 | CCNB1 |
|  |  | Plasminogen activator inhibitor 1 | SERPINE1 |
|  |  | Interferon gamma | IFNG |
|  |  | Interleukin-4 | IL4 |
|  |  | NF-kappa-B essential modulator | IKBKG |
|  |  | 26S proteasome non-ATPase regulatory subunit 3 | PSMD3 |
|  |  | Solute carrier family 2, facilitated glucose transporter member 4 | SLC2A4 |
|  |  | Insulin receptor | INSR |
|  |  | CD40 ligand | CD40LG |
|  |  | Cytochrome c | CYCS |
|  |  | CASP8 and FADD-like apoptosis regulator | CFLAR |
|  |  | Alpha- and gamma-adaptin-binding protein p34 | AAGAB |
|  |  | Insulin | INS |
|  |  | Low affinity immunoglobulin epsilon Fc receptor | FCER2 |
|  |  | Interleukin-13 | IL13 |
|  |  | High affinity immunoglobulin epsilon receptor subunit beta | MS4A2 |
|  |  | Intestinal-type alkaline phosphatase | ALPI |
|  |  | Proteasome activator complex subunit 3 | PSME3 |
|  |  | Glucose-6-phosphatase | G6PC |
|  |  | Adenomatous polyposis coli protein | APC |
|  |  | Transient receptor potential cation channel subfamily M member 2 | TRPM2 |
|  |  | Aldo-keto reductase family 1 member C3 | AKR1C3 |
|  |  | Sodium/iodide cotransporter | SLC5A5 |
|  |  | Sodium/potassium-transporting ATPase subunit gamma | FXYD2 |
|  |  | Dolichyl-phosphate beta-glucosyltransferase | ALG5 |
| 11 | Naringenin | Prostaglandin G/H synthase 1 | PTGS1 |
|  |  | Estrogen receptor | ESR1 |
|  |  | Prostaglandin G/H synthase 2 | PTGS2 |
|  |  | Transcription factor p65 | RELA |
|  |  | RAC-alpha serine/threonine-protein kinase | AKT1 |
|  |  | Apoptosis regulator Bcl-2 | BCL2 |
|  |  | Mitogen-activated protein kinase 3 | MAPK3 |
|  |  | Mitogen-activated protein kinase 1 | MAPK1 |
|  |  | Caspase-3 | CASP3 |
|  |  | Fatty acid synthase | FASN |
|  |  | Low-density lipoprotein receptor | LDLR |
|  |  | Superoxide dismutase [Cu-Zn] | SOD1 |
|  |  | Catalase | CAT |
|  |  | Peroxisome proliferator-activated receptor gamma | PPARG |
|  |  | Microsomal triglyceride transfer protein large subunit | MTTP |
|  |  | Apolipoprotein B-100 | APOB |
|  |  | Phospholipase B1, membrane-associated | PLB1 |
|  |  | 3-hydroxy-3-methylglutaryl-coenzyme A reductase | HMGCR |
|  |  | Glutathione S-transferase P | GSTP1 |
|  |  | UDP-glucuronosyltransferase 1-1 | UGT1A1 |
|  |  | Peroxisome proliferator-activated receptor alpha | PPARA |
|  |  | Sterol regulatory element-binding protein 1 | SREBF1 |
|  |  | Glutathione reductase, mitochondrial | GSR |
|  |  | Multidrug resistance-associated protein 1 | ABCC1 |
|  |  | Adiponectin | ADIPOQ |
|  |  | Sterol O-acyltransferase 2 | SOAT2 |
|  |  | Aldo-keto reductase family 1 member C1 | AKR1C1 |
|  |  | Aspartate aminotransferase, cytoplasmic | GOT1 |
|  |  | 4-aminobutyrate aminotransferase, mitochondrial | ABAT |
|  |  | Liver carboxylesterase 1 | CES1 |
|  |  | Sterol O-acyltransferase 1 | SOAT1 |
| 12 | Eriodictyol | Prostaglandin G/H synthase 1 | PTGS1 |
|  |  | Prostaglandin G/H synthase 2 | PTGS2 |
|  |  | Nuclear receptor coactivator 2 | NCOA2 |
|  |  | Heme oxygenase 1 | HMOX1 |
|  |  | Nuclear factor erythroid 2-related factor 2 | NFE2L2 |
|  |  | NAD(P)H dehydrogenase [quinone] 1 | NQO1 |
| 13 | Acacetin | Nitric oxide synthase, inducible | NOS2 |
|  |  | Prostaglandin G/H synthase 1 | PTGS1 |
|  |  | Androgen receptor | AR |
|  |  | Prostaglandin G/H synthase 2 | PTGS2 |
|  |  | Trypsin-1 | PRSS1 |
|  |  | Nuclear receptor coactivator 2 | NCOA2 |
|  |  | Nuclear receptor coactivator 1 | NCOA1 |
|  |  | Serine/threonine-protein kinase Chk1 | CHEK1 |
|  |  | Beta-2 adrenergic receptor | ADRB2 |
|  |  | Transcription factor p65 | RELA |
|  |  | Apoptosis regulator Bcl-2 | BCL2 |
|  |  | Cyclin-dependent kinase inhibitor 1 | CDKN1A |
|  |  | Apoptosis regulator BAX | BAX |
|  |  | Caspase-3 | CASP3 |
|  |  | Cellular tumor antigen p53 | TP63 |
|  |  | Caspase-8 | CASP8 |
|  |  | Fatty acid synthase | FASN |
|  |  | Tumor necrosis factor ligand superfamily member 6 | FASLG |
| 14 | 7-O-Methylluteolin | Nitric oxide synthase, inducible | NOS2 |
|  |  | Prostaglandin G/H synthase 1 | PTGS1 |
|  |  | Prostaglandin G/H synthase 2 | PTGS2 |
|  |  | Trypsin-1 | PRSS1 |
|  |  | Nuclear receptor coactivator 2 | NCOA2 |
| 15 | Hesperetin | Prostaglandin G/H synthase 1 | PTGS1 |
|  |  | Sodium channel protein type 5 subunit alpha | SCN5A |
|  |  | Prostaglandin G/H synthase 2 | PTGS2 |
|  |  | Nuclear receptor coactivator 2 | NCOA2 |
|  |  | Nuclear receptor coactivator 1 | NCOA1 |
| 16 | Limonin | Cytochrome P450 3A4 | CYP3A4 |
| 17 | Palmitic acid | Cathepsin D | CTSD |
|  |  | Alcohol dehydrogenase 1B | ADH1B |
|  |  | Alcohol dehydrogenase 1C | ADH1C |
|  |  | Prostaglandin G/H synthase 1 | PTGS1 |
|  |  | Prostaglandin G/H synthase 2 | PTGS2 |
|  |  | Rhodopsin | RHO |
|  |  | Nuclear receptor coactivator 2 | NCOA2 |
|  |  | Apoptosis regulator Bcl-2 | BCL2 |
|  |  | Interleukin-10 | IL10 |
|  |  | Tumor necrosis factor | TNFSF15 |
|  |  | Collagen alpha-1(I) chain | COL1A1 |
|  |  | Solute carrier family 22 member 5 | SLC22A5 |
|  |  | Choline-phosphate cytidylyltransferase A | PCYT1A |
| 18 | Linoleic acid | Prostaglandin G/H synthase 1 | PTGS1 |
|  |  | Prostaglandin G/H synthase 2 | PTGS2 |
|  |  | Retinoic acid receptor RXR-alpha | RXRA |
|  |  | Nuclear receptor coactivator 2 | NCOA2 |
|  |  | Lysozyme | LYZ |
|  |  | Sodium-dependent noradrenaline transporter | SLC6A2 |
|  |  | Gamma-aminobutyric acid receptor subunit alpha-1 | GABRA1 |
|  |  | Transient receptor potential cation channel subfamily V member 1 | TRPV1 |
|  |  | Muscarinic acetylcholine receptor M1 | CHRM1 |
|  |  | Muscarinic acetylcholine receptor M2 | CHRM2 |
| 19 | Rutin | Transcription factor p65 | RELA |
|  |  | Tumor necrosis factor | TNFSF15 |
|  |  | Interleukin-6 | IL6 |
|  |  | Caspase-3 | CASP3 |
|  |  | NADPH--cytochrome P450 reductase | POR |
|  |  | Superoxide dismutase [Cu-Zn] | SOD1 |
|  |  | Catalase | CAT |
|  |  | Interleukin-1 beta | IL1B |
|  |  | Interleukin-8 | CXCL8 |
|  |  | Protein kinase C beta type | PRKCB |
|  |  | Arachidonate 5-lipoxygenase | ALOX5 |
|  |  | 3-hydroxy-3-methylglutaryl-coenzyme A reductase | HMGCR |
|  |  | Hyaluronan synthase 2 | HAS2 |
|  |  | Glutathione S-transferase P | GSTP1 |
|  |  | Type I iodothyronine deiodinase | DIO1 |
|  |  | Insulin | INS |
|  |  | Low affinity immunoglobulin epsilon Fc receptor | FCER2 |
|  |  | Integrin beta-2 | ITGB2 |
|  |  | Thromboxane A2 receptor | TBXA2R |
| 20 | 4',5,7-Trimethoxyflavone | Nitric oxide synthase, inducible | NOS2 |
|  |  | Prostaglandin G/H synthase 1 | PTGS1 |
|  |  | Sodium channel protein type 5 subunit alpha | SCN5A |
|  |  | Prostaglandin G/H synthase 2 | PTGS2 |
|  |  | Alpha-2C adrenergic receptor | ADRA2C |
|  |  | Alpha-1B adrenergic receptor | ADRA1B |
|  |  | Beta-2 adrenergic receptor | ADRB2 |
|  |  | Estrogen receptor beta | ESR2 |
|  |  | Trypsin-1 | PRSS1 |
|  |  | Nuclear receptor coactivator 2 | NCOA2 |
| 21 | 3,4'-Di-O-methylkaempferol | Nitric oxide synthase, inducible | NOS2 |
|  |  | Prostaglandin G/H synthase 1 | PTGS1 |
|  |  | Androgen receptor | AR |
|  |  | Sodium channel protein type 5 subunit alpha | SCN5A |
|  |  | Prostaglandin G/H synthase 2 | PTGS2 |
|  |  | Retinoic acid receptor RXR-alpha | RXRA |
|  |  | Mitogen-activated protein kinase 14 | MAPK14 |
|  |  | Glycogen synthase kinase-3 beta | GSK3B |
|  |  | Serine/threonine-protein kinase Chk1 | CHEK1 |
|  |  | Trypsin-1 | PRSS1 |
| 22 | Tangeretin | Nitric oxide synthase, inducible | NOS2 |
|  |  | Prostaglandin G/H synthase 1 | PTGS1 |
|  |  | Potassium voltage-gated channel subfamily H member 2 | KCNH2 |
|  |  | Androgen receptor | AR |
|  |  | Sodium channel protein type 5 subunit alpha | SCN5A |
|  |  | Prostaglandin G/H synthase 2 | PTGS2 |
|  |  | Coagulation factor VII | F7 |
|  |  | Acetylcholinesterase | ACHE |
|  |  | Alpha-1B adrenergic receptor | ADRA1B |
|  |  | Beta-2 adrenergic receptor | ADRB2 |
|  |  | Estrogen receptor beta | ESR2 |
|  |  | Serine/threonine-protein kinase Chk1 | CHEK1 |
|  |  | Trypsin-1 | PRSS1 |
|  |  | Nuclear receptor coactivator 2 | NCOA2 |
|  |  | Cyclin-dependent kinase inhibitor 1 | CDKN1A |
|  |  | Eukaryotic translation initiation factor 6 | EIF6 |
|  |  | Heme oxygenase 1 | HMOX1 |
|  |  | Cytochrome P450 1A1 | CYP1A1 |
| 23 | 3,5,7,8,3',4'-Hexamethoxyflavone | Nitric oxide synthase, inducible | NOS2 |
|  |  | Estrogen receptor | ESR1 |
|  |  | Androgen receptor | AR |
|  |  | Sodium channel protein type 5 subunit alpha | SCN5A |
|  |  | Peroxisome proliferator activated receptor gamma | PPARG |
|  |  | Prostaglandin G/H synthase 2 | PTGS2 |
|  |  | Coagulation factor VII | F7 |
|  |  | Estrogen receptor beta | ESR2 |
|  |  | Glycogen synthase kinase-3 beta | GSK3B |
|  |  | Serine/threonine-protein kinase Chk1 | CHEK1 |
|  |  | Trypsin-1 | PRSS1 |
|  |  | Nuclear receptor coactivator 2 | NCOA2 |
| 24 | 3,5,6,7,8,3',4'-Heptamethoxyflavone | Potassium voltage-gated channel subfamily H member 2 | KCNH2 |
|  |  | Estrogen receptor | ESR1 |
|  |  | Androgen receptor | AR |
|  |  | Prostaglandin G/H synthase 2 | PTGS2 |
|  |  | Coagulation factor VII | F7 |
|  |  | Estrogen receptor beta | ESR2 |
|  |  | Glycogen synthase kinase-3 beta | GSK3B |
|  |  | Nuclear receptor coactivator 2 | NCOA2 |
| 25 | Atractylenolide Ⅰ | Gamma-aminobutyric acid receptor subunit alpha-1 | GABRA1 |
|  |  | Vascular endothelial growth factor A | VEGFA |
|  |  | Tumor necrosis factor | TNFSF15 |
|  |  | Interleukin-6 | IL6 |
|  |  | Interleukin-1 beta | IL1B |
|  |  | Dopamine D2 receptor | DRD2 |
|  |  | Estrogen receptor | ESR1 |
|  |  | Estrogen receptor beta | ESR2 |
|  |  | Insulin | INS |
|  |  | Insulin receptor | INSR |
|  |  | Placenta growth factor | PGF |
| 26 | Atractylenolide Ⅲ | Gamma-aminobutyric acid receptor subunit alpha-1 | GABRA1 |
|  |  | Glutamate receptor 2 | GRIA2 |
|  |  | Tumor necrosis factor | TNFSF15 |
|  |  | Estrogen receptor | ESR1 |
|  |  | Estrogen receptor beta | ESR2 |
|  |  | Insulin | INS |
|  |  | Insulin receptor | INSR |
|  |  | Dopamine D2 receptor | DRD2 |
| 27 | Atractylone | Muscarinic acetylcholine receptor M3 | CHRM3 |
|  |  | Muscarinic acetylcholine receptor M1 | CHRM1 |
|  |  | Beta-1 adrenergic receptor | ADRB1 |
|  |  | Sodium channel protein type 5 subunit alpha | SCN5A |
|  |  | Muscarinic acetylcholine receptor M4 | CHRM4 |
|  |  | Sodium-dependent noradrenaline transporter | SLC6A2 |
|  |  | Alpha-1A adrenergic receptor | ADRA1A |
|  |  | Muscarinic acetylcholine receptor M2 | CHRM2 |
|  |  | Alpha-1B adrenergic receptor | ADRA1B |
|  |  | Sodium-dependent dopamine transporter | SLC6A3 |
|  |  | Beta-2 adrenergic receptor | ADRB2 |
|  |  | Mu-type opioid receptor | OPRM1 |
|  |  | Estrogen receptor | ESR1 |
|  |  | Estrogen receptor beta | ESR2 |
|  |  | Insulin | INS |
|  |  | Insulin receptor | INSR |
|  |  | Dopamine D2 receptor | DRD2 |
| 28 | 3β-acetoxyatractylone | Muscarinic acetylcholine receptor M3 | CHRM3 |
|  |  | Muscarinic acetylcholine receptor M1 | CHRM1 |
|  |  | Androgen receptor | AR |
|  |  | Sodium channel protein type 5 subunit alpha | SCN5A |
|  |  | Prostaglandin G/H synthase 2 | PTGS2 |
|  |  | Retinoic acid receptor RXR-alpha | RXRA |
|  |  | Acetylcholinesterase | ACHE |
|  |  | Alpha-1A adrenergic receptor | ADRA1A |
|  |  | Muscarinic acetylcholine receptor M2 | CHRM2 |
|  |  | Beta-2 adrenergic receptor | ADRB2 |
|  |  | Mu-type opioid receptor | OPRM1 |
|  |  | Gamma-aminobutyric acid receptor subunit alpha-1 | GABRA1 |
| 29 | Selina-4(14),7(11)-dien-8-one | Muscarinic acetylcholine receptor M3 | CHRM3 |
|  |  | Muscarinic acetylcholine receptor M1 | CHRM1 |
|  |  | Prostaglandin G/H synthase 2 | PTGS2 |
|  |  | Sodium-dependent noradrenaline transporter | SLC6A2 |
|  |  | Gamma-aminobutyric acid receptor subunit alpha-1 | GABRA1 |
|  |  | Muscarinic acetylcholine receptor M2 | CHRM2 |
| 30 | 8β-ethoxyasterolid | Prostaglandin G/H synthase 2 | PTGS2 |
|  |  | Gamma-aminobutyric acid receptor subunit alpha-1 | GABRA1 |
|  |  | Nuclear receptor coactivator 2 | NCOA2 |
|  |  | Nuclear receptor coactivator 1 | NCOA1 |
| 31 | Synephrine | Neuromedin-U receptor 2 | NMUR2 |
|  |  | Alpha-1a adrenergic receptor | ADRA1A |
|  |  | Beta-1 adrenergic receptor | ADRB1 |
|  |  | Alpha-1b adrenergic receptor | ADRA1B |
|  |  | Alpha-2a adrenergic receptor | ADRA2A |
|  |  | Alpha-2b adrenergic receptor | ADRA2B |
|  |  | Adrenergic receptor beta | ADRB2 |
|  |  | Adrenergic receptor alpha-2 | ADRA2C |
|  |  | Dopamine D2 receptor | DRD2 |
|  |  | Alpha-1d adrenergic receptor | ADRA1D |
|  |  | Mu opioid receptor | OPRM1 |
|  |  | Serotonin 3a | HTR3A |
|  |  | Beta-3 adrenergic receptor | ADRB3 |
|  |  | Dopamine D4 receptor | DRD4 |
|  |  | Dopamine D3 receptor | DRD3 |
|  |  | Serotonin transporter | SLC6A4 |
| 32 | Atractylenolide Ⅱ | Androgen receptor | AR |
|  |  | Muscarinic acetylcholine receptor M3 | CHRM3 |
|  |  | 3-hydroxy-3-methylglutaryl-coenzyme A reductase | HMGCR |
|  |  | Interleukin-1 beta | IL1B |
|  |  | Progesterone receptor | PGR |
|  |  | Peroxisome proliferator-activated receptor gamma | PPARG |
|  |  | Dopamine D2 receptor | DRD2 |
|  |  | Gamma-aminobutyric acid receptor subunit alpha-1 | GABRA1 |
|  |  | Estrogen receptor | ESR1 |
|  |  | Estrogen receptor beta | ESR2 |
|  |  | Insulin | INS |
|  |  | Insulin receptor | INSR |
|  |  | Glutamate receptor 2 | GRIA2 |

Table S7. The overlapping targets of PAMR and FD

| No. | Compounds | Targets |
| --- | --- | --- |
| 1 | Aesculin | PTGS2 |
| 2 | 4-caffeoylquinic acid | PTGS2 |
| 3 | 3-O-p-Coumaroylquinic acid | PTGS1/PTGS2 |
| 4 | Naringin | CDKN1A/TNFSF15/RAF1 |
| 5 | Diosmin | PTGS1/PTGS2/PRSS1 |
| 6 | Hesperidin | BAX/CASP3/PTGS2/ICAM1/HTR3A |
| 7 | Isovitexin | PTGS2/AR/RELA/IKBKB/TNFSF15 |
| 8 | Vitexin | AR/PTGS2 |
| 9 | Naringenin 7-O-glucoside | PTGS2 |
| 10 | Apigenin | PTGS1/AR/PTGS2/PRSS1/F7/RELA/AKT1/VEGFA/CCND1/BCL2/BCL2L1/FOS/CDKN1A/BAX/CASP9/PLAU/MMP9/RB1/TNFSF15/JUN/CASP3/TP63/NFKBIA/ODC1/MDM2/MMP1/HIF1A/IGF1R/RUNX1T1/HMOX1/ICAM1/MCL1/CCND2/IL2/CCNB1/SERPINE1/IFNG/IL4/IKBKG/SLC2A4/INSR/CD40LG/CYCS/CFLAR/INS/FCER2/IL13/G6PC/APC/FXYD2 |
| 11 | Naringenin | PTGS1/ESR1/PTGS2/RELA/AKT1/BCL2/MAPK3/MAPK1/CASP3/FASN/LDLR/SOD1/CAT/PPARG/MTTP/APOB/HMGCR/GSTP1/UGT1A1/PPARA/SREBF1/ABCC1/ADIPOQ |
| 12 | Eriodictyol | PTGS1/PTGS2/HMOX1/NFE2L2/NQO1 |
| 13 | Acacetin | NOS2/PTGS1/AR/PTGS2/PRSS1/CHEK1/ADRB2/RELA/BCL2/CDKN1A/BAX/CASP3/TP63/CASP8/FASN/FASLG |
| 14 | 7-O-Methylluteolin | NOS2/PTGS1/PTGS2/PRSS1 |
| 15 | Hesperetin | PTGS1/PTGS2 |
| 16 | Limonin | CYP3A4 |
| 17 | Palmitic acid | CTSD/ADH1B/ADH1C/PTGS1/PTGS2/RHO/BCL2/IL10/TNFSF15/SLC22A5 |
| 18 | Linoleic acid | PTGS1/PTGS2/RXRA/TRPV1 |
| 19 | Rutin | RELA/TNFSF15/IL6/CASP3/SOD1/CAT/IL1B/CXCL8/PRKCB/ALOX5/HMGCR/GSTP1/INS/FCER2 |
| 20 | 4',5,7-Trimethoxyflavone | NOS2/PTGS1/PTGS2/ADRA2C/ADRB2/ESR2/PRSS1 |
| 21 | 3,4'-Di-O-methylkaempferol | NOS2/PTGS1/AR/PTGS2/RXRA/MAPK14/GSK3B/CHEK1/PRSS1 |
| 22 | Tangeretin | NOS2/PTGS1/AR/PTGS2/F7/ACHE/ADRB2/ESR2/CHEK1/PRSS1/CDKN1A/HMOX1/CYP1A1 |
| 23 | 3,5,7,8,3',4'-Hexamethoxyflavone | NOS2/ESR1/AR/PPARG/PTGS2/F7/ESR2/GSK3B/CHEK1/PRSS1 |
| 24 | 3,5,6,7,8,3',4'-Heptamethoxyflavone | ESR1/AR/PTGS2/F7/ESR2/GSK3B |
| 25 | Atractylenolide Ⅰ | VEGFA/TNFSF15/IL6/IL1B/DRD2/ESR1/ESR2/INS/INSR |
| 26 | Atractylenolide Ⅲ | TNFSF15/DRD2/ESR1/ESR2/INS/INSR |
| 27 | Atractylone | CHRM3/SLC6A3/ADRB2/OPRM1/DRD2/ESR1/ESR2/INS/INSR |
| 28 | 3β-acetoxyatractylone | CHRM3/AR/PTGS2/RXRA/ACHE/ADRB2/OPRM1 |
| 29 | selina-4(14),7(11)-dien-8-one | CHRM3/PTGS2 |
| 30 | 8β-ethoxyasterolid | PTGS2 |
| 31 | Synephrine | ADRA2A/ADRA2B/ADRB2/ADRA2C/DRD2/OPRM1/HTR3A/SLC6A4 |
| 32 | Atractylenolide Ⅱ | AR/CHRM3/HMGCR/IL1B/PGR/PPARG/ESR1/ESR2/INS/INSR |


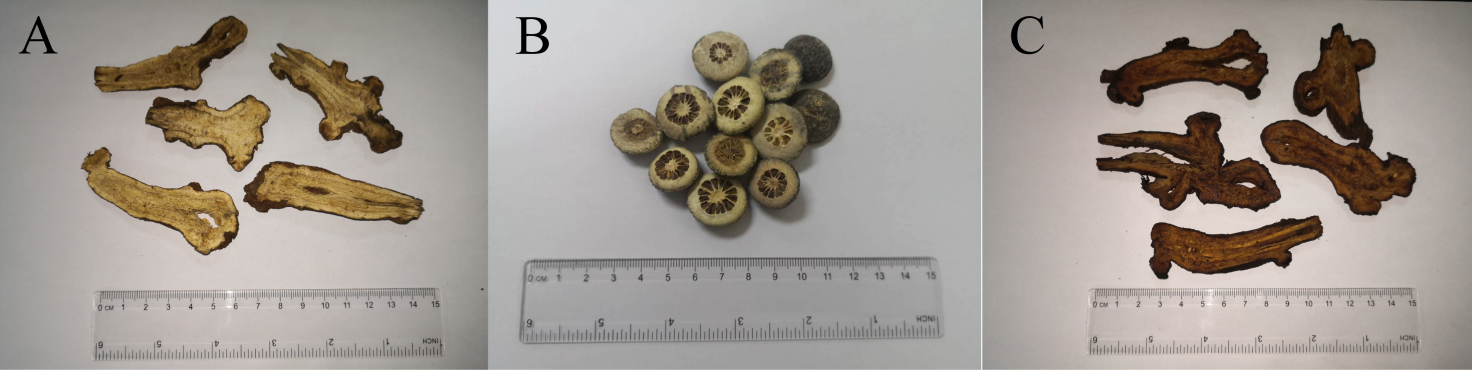


**Figure S1.** Appearance of the samples

RAMR (A); RAFI (B); PAMR (C)


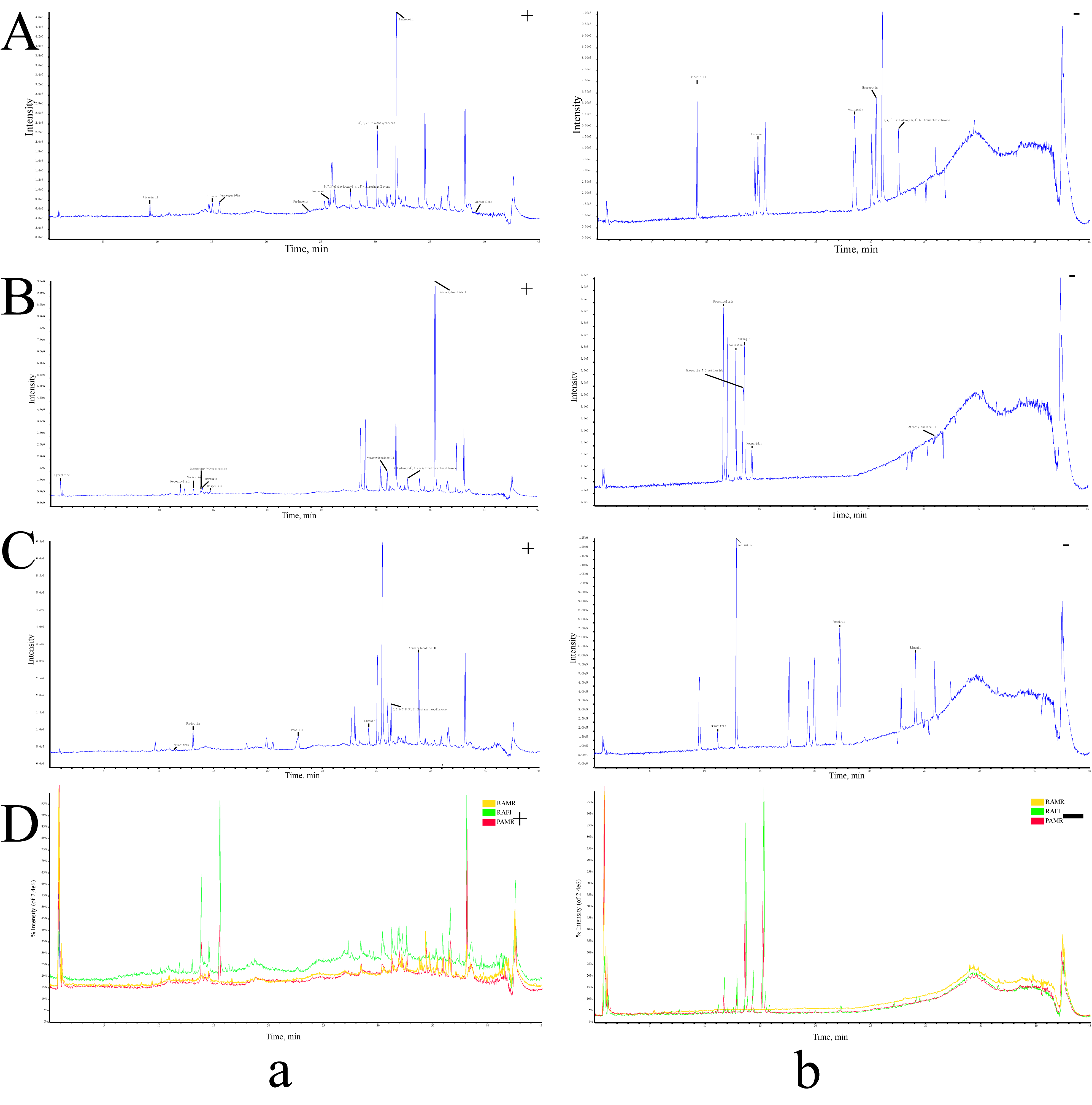


**Figure S2.** TICs of the standard compounds and samples in positive (a) and negative ions (b), based on UPLC-Q-TOF-MS/MS

**A: standard group 1:** Naringenin, Hesperetin, 4',5,7-Trimethoxyflavone, 5,7,3'-Trihydroxy-6,4',5'-Trimethoxyflavone, Tangeretin, Diosmin, Neohesperidin, Atractylone and Vicenin Ⅱ

**B: standard group 2:** 5-Hydroxy-3',4',6,7,8-pentamethoxyflavone, Narirutin, Neoeriocitrin, Naringenin-7-O-glucoside, Naringin, Hesperidin, Synephrine, Atractylenolide Ⅰ and Atractylenolide Ⅲ

**C: standard group 3:** 3,3',4',5,6,7,8-Heptamethoxyflavone, Limonin, Eriocitrin, Poncirin and Atractylenolide Ⅱ

**D:** The TICs of RAMR, RAFI and PAMR


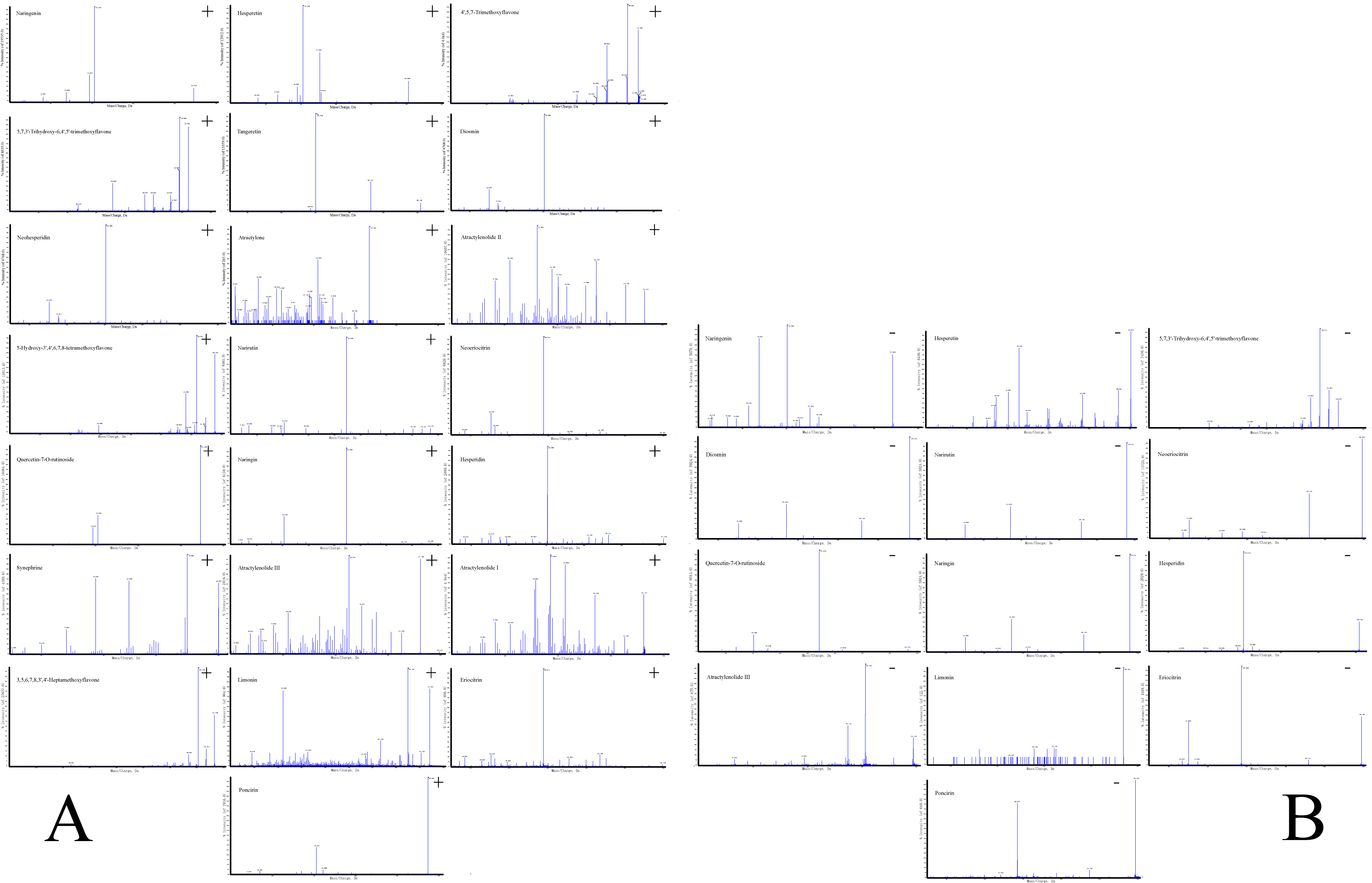


**Figure S3** The mass spectra properties of the standard compounds in positive (A) and negative ions (B), based on UPLC-Q-TOF-MS/MS
