# Supplementary figures and images for: Rhizoma Atractylodis Macrocephalae—Assessing the influence of herbal processing methods and improved effects on functional dyspepsia
Source: Front Pharmacol. 2023 Aug 4;14:1236656. doi: 10.3389/fphar.2023.1236656 (PMC10436233; doi:10.3389/fphar.2023.1236656)

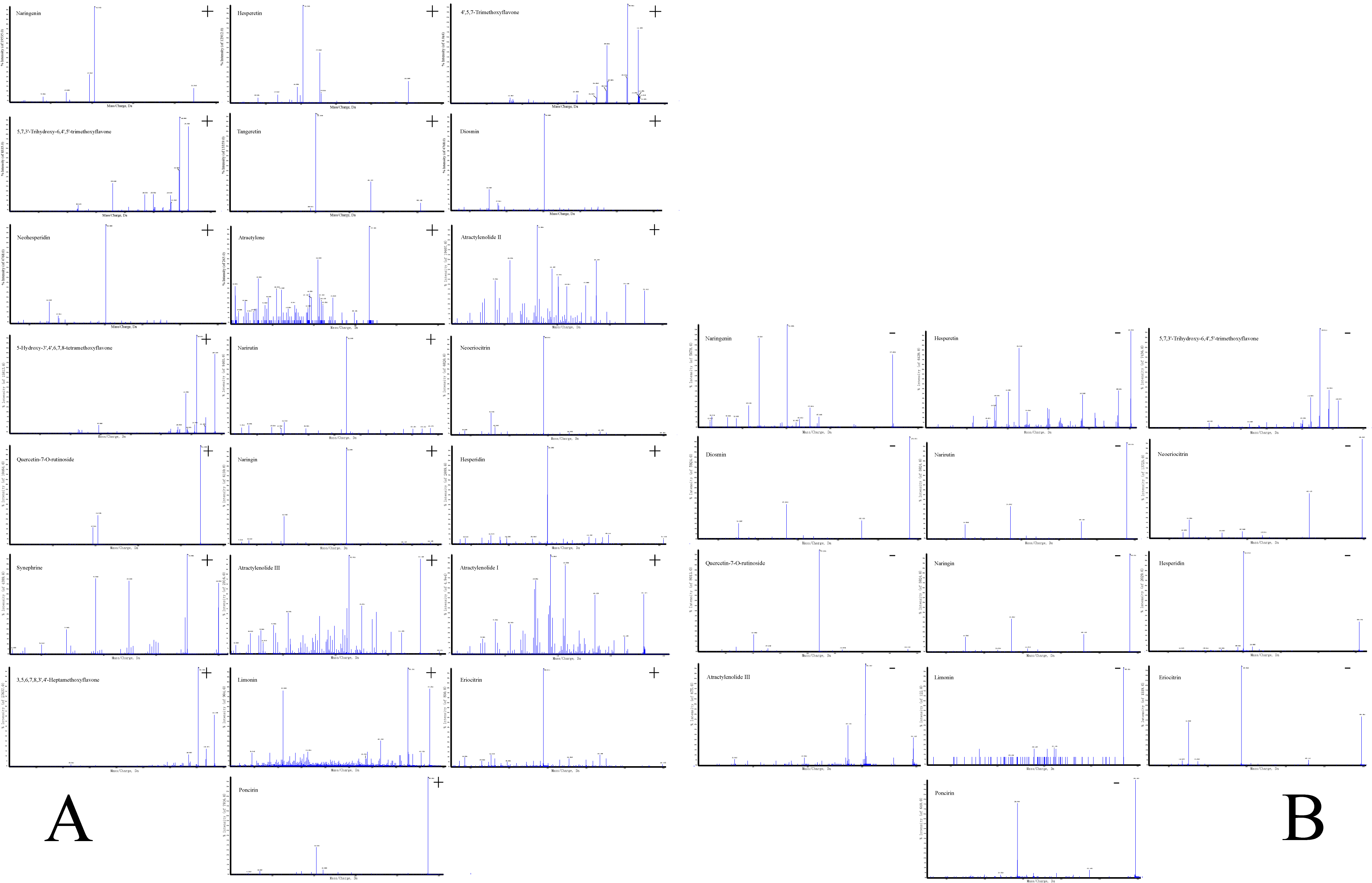

Supplement: Supplementary file 2 [file Image3.TIF]

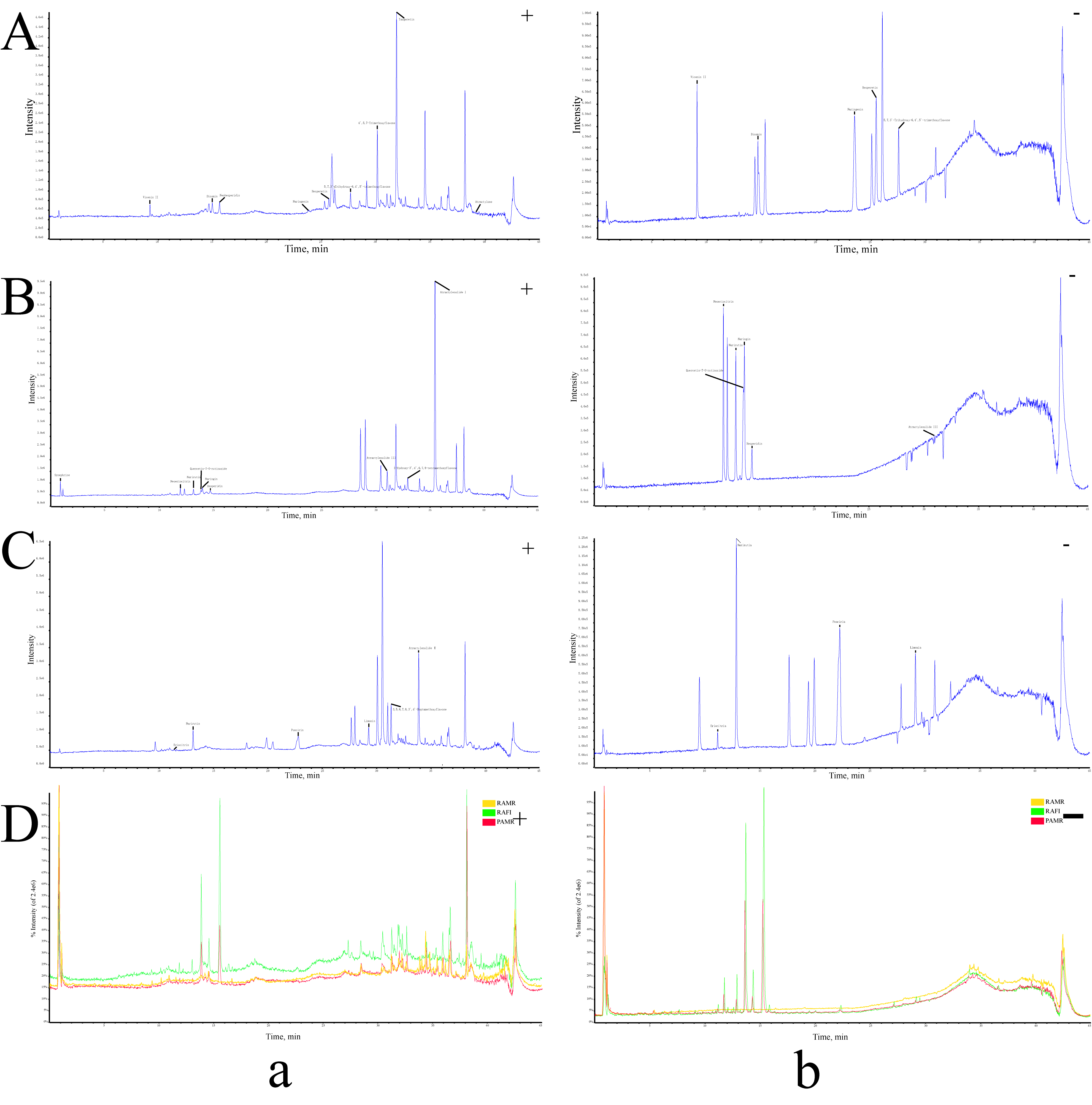

Supplement: Supplementary file 3 [file Image2.TIF]

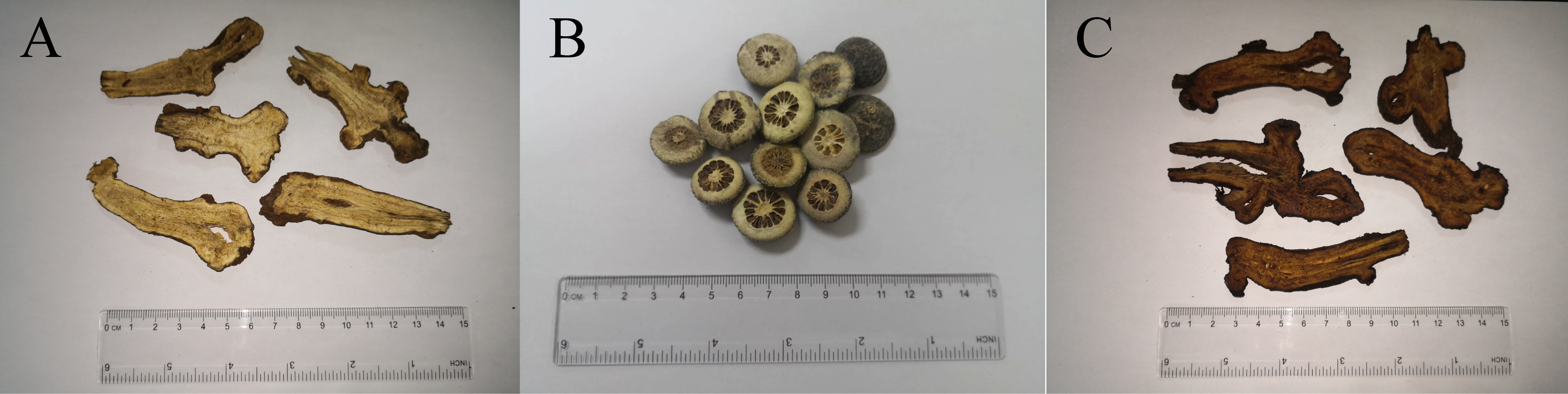

Supplement: Supplementary file 4 [file Image1.TIF]
